# Precursor Self‐Assembly Identified as a General Pathway for Colloidal Semiconductor Magic‐Size Clusters

**DOI:** 10.1002/advs.201800632

**Published:** 2018-10-23

**Authors:** Linxi Wang, Juan Hui, Junbin Tang, Nelson Rowell, Baowei Zhang, Tingting Zhu, Meng Zhang, Xiaoyu Hao, Hongsong Fan, Jianrong Zeng, Shuo Han, Kui Yu

**Affiliations:** ^1^ Institute of Atomic and Molecular Physics Sichuan University Chengdu 610065 P. R. China; ^2^ National Research Council of Canada Ottawa Ontario K1A 0R6 Canada; ^3^ Engineering Research Center in Biomaterials Sichuan University Chengdu 610065 P. R. China; ^4^ Shanghai Synchrotron Radiation Facility Shanghai Institute of Applied Physics Chinese Academy of Sciences Shanghai 201204 P. R. China; ^5^ School of Chemical Engineering Sichuan University Chengdu 610065 P. R. China

**Keywords:** colloidal semiconductor nanocrystals, dense phase reactions, magic‐size clusters (MSCs), precursor self‐assembly, self‐assembly, two‐step approach

## Abstract

Little is known about the formation pathway of colloidal semiconductor magic‐size clusters (MSCs). Here, the synthesis of the first single‐ensemble ZnSe MSCs, which exhibit a sharp optical absorption singlet peaking at 299 nm, is reported; their formation is independent of Zn and Se precursors used. It is proposed that the formation of MSCs starts with precursor self‐assembly followed by Zn and Se covalent bond formation to result in immediate precursors (IPs) which can transform into the MSCs. It is demonstrated that the IPs in cyclohexane appear transparent in optical absorption, and become visible as MSCs exhibiting one sharp optical absorption peak when a primary amine is added at room temperature. It is shown that when the preparation of the IP is controlled to be within the induction period, which occurs prior to nucleation and growth of conventional quantum dots (QDs), the resulting MSCs can be produced without the complication of the simultaneous coproduction of conventional QDs. The present study reveals the existence of precursor self‐assembly which leads to the formation of colloidal semiconductor MSCs and provides insights into a multistep nucleation process in cluster science.

## 1 Introduction

Cluster science is dedicated to bridge the gap of the evolution of fundamental properties with size from individual molecules toward extended bulk materials, while self‐assembly of small building blocks has been demonstrated to generate larger structures.^1^, ^2^, ^3^, ^4^, ^5^, ^6^, ^7^, ^8^ Crystallization is a unique case of self‐assembly, occurring frequently in nature and being vital for many applications including pharmaceutical and chemical manufacturing. Both the classical and modern nucleation models, which are one‐step and multistep based, respectively, engage the concept of self‐assembly.^9^, ^10^, ^11^, ^12^, ^13^, ^14^, ^15^, ^16^, ^17^, ^18^

Colloidal semiconductor magic‐size clusters (MSCs) are essential to our understanding of the evolution of fundamental properties from molecules to quantum dots (QDs). Semiconductor QDs have emerged as a special class of functional nanomaterials and have attracted a great deal of attention for more than two decades.^19^, ^20^, ^21^, ^22^, ^23^, ^24^, ^25^, ^26^, ^27^, ^28^, ^29^, ^30^, ^31^, ^32^, ^33^, ^34^, ^35^ Interestingly, the current effort on semiconductor nanocrystals (NCs) has marched heavily into application‐oriented research, with limited attention paid to the synthesis of MSCs in a single‐ensemble form without the coexistence of other‐size NCs.^36^, ^37^, ^38^, ^39^, ^40^ Compared with conventional QDs, MSCs are usually characterized by much narrower optical absorption bands, because of their very tight size distributions.^41^, ^42^, ^43^ Conventional QDs are sometimes labeled as regular QDs (RQDs) to differentiate them from MSCs. The narrow size distribution of MSCs is a direct result of their morphology with particular stability. The presence of MSCs has been associated with the occurrence of sharp peaks at persistent positions in optical absorption.^43^, ^44^, ^45^, ^46^, ^47^ The reactions involved in the formation of M_2_E*_n_* RQDs and MSCs and monomers (where M represents monovalent to trivalent cations and E stands for chalcogenides) have been established to be the same, based on the same phosphorus‐containing products monitored by ^31^P NMR.^25^, ^26^, ^27^, ^28^, ^29^ However, little has been known about the formation pathway of MSCs.

We report in the present work an elaboration regarding the precursor self‐assembly that drives the formation of MSCs. Based on the concept that the development of MSCs can occur preferentially through a structural transformation from their immediate precursors (IPs) following first‐order unimolecular reaction kinetics,^38^, ^39^ we applied an approach which consists of two decoupled steps to synthesize MSCs. The first step was to prepare IPs at a relatively high temperature (prior to the formation of RQDs); the second step was to transfer IPs to MSCs at room temperature. With zinc selenide (ZnSe) as a model system, different Zn and Se precursors were studied. The resulting ZnSe MSCs are referred to as MSC‐299, a symbol derived from their optical absorption characteristics. For the IPs of the MSCs, they are accordingly labeled as IP‐299, which are transparent in cyclohexane in optical absorption. For the following reactions with various Zn and Se precursors, the detection of MSC‐299 will be addressed

(1) $$\mathrm{Zn}{\left(\mathrm{OA}\right)}_{2}+\mathrm{SeTOP}\Rightarrow \mathrm{IP–}299\Rightarrow \mathrm{MSC–}299$$

(2) $$\mathrm{Zn}{\left(\mathrm{OA}\right)}_{2}+\mathrm{SeODE}\Rightarrow \mathrm{IP–}299\Rightarrow \mathrm{MSC–}299$$

(3) $$\mathrm{Zn}{\left(\mathrm{OA}\right)}_{2}+{\mathrm{SePPh}}_{2}H\Rightarrow \mathrm{IP–}299\Rightarrow \mathrm{MSC–}299$$

(4) $$\mathrm{Zn}{\left(\mathrm{OA}\right)}_{2}+\mathrm{SeTOP}+{\mathrm{HPPh}}_{2}\Rightarrow \mathrm{IP–}299\Rightarrow \mathrm{MSC–}299$$

(5) $$\mathrm{Zn}{\left(\mathrm{OAc}\right)}_{2}\mathrm{/OLA}+\mathrm{SeTOP}+{\mathrm{HPPh}}_{2}\Rightarrow \mathrm{IP–}299\Rightarrow \mathrm{MSC–}299$$

(6) $$\mathrm{Zn}{\left(\mathrm{OAc}\right)}_{2}\mathrm{/OLA}+\mathrm{SeTOP}\Rightarrow \mathrm{RQDs}$$

For Reactions (1) through (4) when zinc oleate (Zn(OA)_2_, Zn(OOCC_17_H_33_)_2_) was used as a Zn precursor, the first step was performed in 1‐octadecene (ODE). For Reactions (5) and (6) when zinc acetate (Zn(OAc)_2_, Zn(OOCCH_3_)_2_) was used in oleylamine (OLA, C_18_H_35_NH_2_) to prepare Zn(OAc)_2_/OLA as a Zn precursor,^38^ the first step was carried out in OLA. Powder selenium (Se) was used as a Se source, with a tertiary phosphine tri‐*n*‐octylphosphine (TOP) and/or a secondary phosphine diphenylphosphine (HPPh_2_), or ODE to promote its reactivity. The two‐step approach, different from hot‐injection and heating‐up approaches,^20^, ^21^, ^22^, ^23^ was validated to be effective in the present case, as it was reported previously.^38^, ^39^ We then conclude that the two‐step approach is generally applicable for the growth of MSCs as a sole ensemble product. Production of crystalline materials with precise control from liquid‐phase precursors has been central to materials science and process engineering. It appears that precursor self‐assembly is independent of the nature of the Zn and Se precursors used and occurs as a general pathway for the formation of MSCs. Our suggested self‐assembly pathway is in agreement with the recent report on the high concentration synthesis of CdS MSCs.^40^ Reactions leading to clusters or aggregates can share a general pathway driven by reactant self‐assembly to occur in dense phases.^2^, ^3^, ^4^, ^5^, ^6^, ^7^, ^8^

## 2 Results and Discussion

For our investigation on the formation pathway of MSCs, we used ZnSe as a model system, exploring the above reactions with experimental conditions optimized for the first step of a two‐step approach to MSC‐299. The optimum method to effectively synthesize ZnSe MSCs as a single product, exhibiting a single and sharp absorption peak, in one step on a routine basis is yet to be determined. For the optical spectra reported,^44^, ^45^, ^46^, ^47^, ^48^ the absorption peaks, which were relatively narrow and/or persistent, occurred at ≈279, 289, 328, and 347 nm. These peaks were assigned as two absorption doublets, one with individual peaks at 279 and 289 nm^44^, ^45^ and the other with individual peaks at 328 and 347 nm.^46^, ^47^, ^48^ Table S1 (Supporting Information) contains a summary of the literature reports regarding the synthesis of colloidal ZnSe NCs.^44^, ^45^, ^46^, ^47^, ^48^, ^49^, ^50^, ^51^, ^52^, ^53^, ^54^, ^55^, ^56^

**Figure 1:**
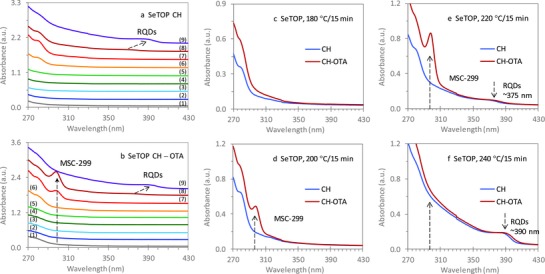
Optical absorption spectroscopy exploring the induction period prior to nucleation and growth of RQDs for the reaction of Zn(OA)_2_ + SeTOP in ODE with a Se concentration of 60 mmol kg^−1^. Samples were extracted after the reaction temperature had been held for 15 min at each of the following nine temperatures (1) 80, (2) 100, (3) 120, (4) 140, (5) 160, (6) 180, (7) 200, (8) 220, and (9) 240 °C; they (15 µL each) were dispersed a) in cyclohexane (CH, 3.0 mL) and b) in a mixture of CH (1.0 mL) and the primary amine OTA (2.0 mL). The absorption spectra collected from the CH (blue traces) and CH–OTA (red traces) dispersions are compared for the c) 180, d) 200, e) 220, and f) 240 °C samples. The induction period is clearly observed to be below 200 °C (red spectra). ZnSe MSC‐299 was detected only in the 200 and 220 °C samples and only when dispersed in the CH–OTA mixture, but not when dispersed in CH.

**Figure 2:**
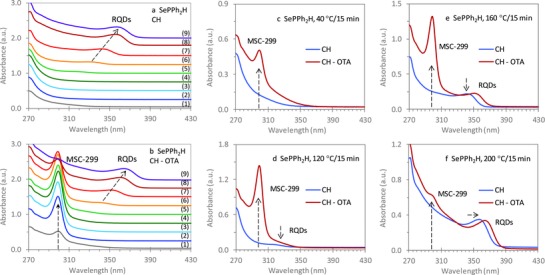
Optical absorption spectroscopy investigating the induction period for the reaction of Zn(OA)_2_ + SePPh_2_H in ODE with a Se concentration of 60 mmol kg^−1^. Samples were extracted after the reaction temperature had been held for 15 min at each of the following nine temperatures (1) 40, (2) 60, (3) 80, (4) 100, (5) 120, (6) 140, (7) 160, (8) 180, and (9) 200 °C. The samples (15 µL each) were dispersed a) in 3.0 mL of CH and b) in a mixture of 2.0 mL of CH and 1.0 mL of OTA. When the temperature was below 120 °C (light green spectrum), the reaction appeared to be in its induction period. We compared the absorption spectra of the two dispersions for samples (15 µL) collected at c) 40, d) 120, e) 160, and f) 200 °C. ZnSe MSC‐299 was detected in the 60—180 °C samples when dispersed in the CH and OTA mixture (red traces), but not in CH (blue traces).

**Figure 3:**
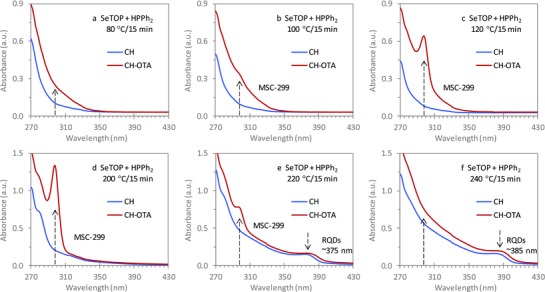
Optical absorption spectroscopy characterizing the induction period for the reaction of Zn(OA)_2_ + SeTOP + HPPh_2_ in ODE with a Se concentration of 60 mmol kg^−1^. Samples were taken 15 min after the temperature was reached, as indicated at a) 80, b) 100, c) 120, d) 200, e) 220, and f) 240 °C. The absorption spectra are compared for the same sample (15 µL) dispersed in the 3.0 mL of CH (blue traces) and in the mixture of 2.0 mL of CH and 1.0 mL of OTA (red traces). The induction period is below 180 °C. ZnSe MSC‐299 was detected in all the samples extracted between 100 and 220 °C and dispersed in the CH–OTA mixture, but not when dispersed in CH.

**Figure 4:**
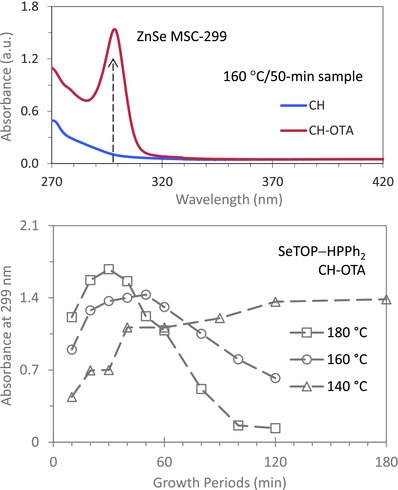
Analysis of our two‐step approach for the reaction of Zn(OA)_2_ and SeTOP with HPPh_2_ with a constant temperature mode instead of a temperature increase mode (for which results are shown in Figure 3 and Figure S3, Supporting Information). To explore the induction period, a constant temperature mode was performed with the temperature held at 140 or 160 or 180 °C. (Top) The absorption spectra of a 50 min sample (15 µL) from the 160 °C batch dispersed in 3.0 mL of CH (blue trace) and in the mixture of 2.0 mL of CH and 1.0 mL of OTA (red trace). (Bottom) The absorbance at 299 nm versus the elapsed reaction time, for the three batch samples (15 µL, first step) and afterward dispersed in the mixture of 2.0 mL CH and 1.0 mL OTA (second step). The first‐step samples were collected at the different growth periods. Remarkably, there is a large experimental window for the optimized formation of ZnSe MSC‐299 from its immediate precursor IP‐299.

**Figure 5:**
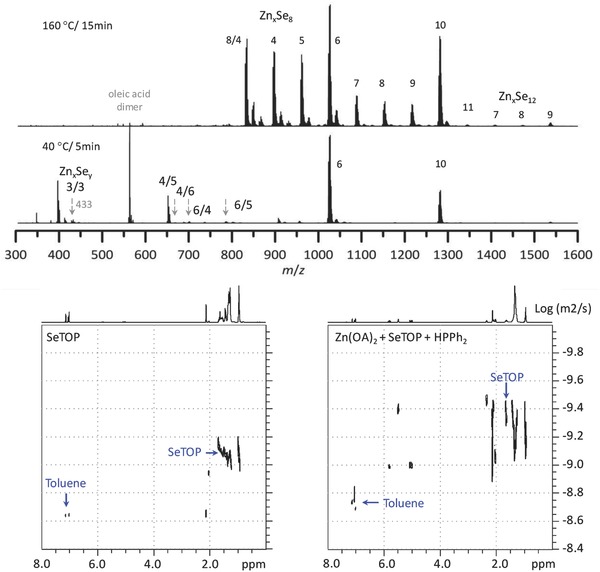
ESI‐MS and DOSY‐NMR studies of Reaction (4). (Top) Mass spectra of the 40 and 160 °C samples (as indicated) from Reaction (4) of Zn(OA)_2_ + SeTOP + HPPh_2_. Figure S5‐1 (Supporting Information) shows the corresponding optical absorption spectra of the same two samples with the growth temperature and period indicated. ZnSe MSC‐299 was not apparently present in the 40 °C sample, but was detected in the 160 °C sample dispersed in the CH–OTA mixture. The detection of the Zn*_x_*Se*_y_* fragments suggests that the formation of Zn and Se covalent bonds took place even at 40 °C. (Bottom) ^1^H NMR DOSY spectra of (left) 5.0 mg SeTOP and (right) a mixture prepared with 33.5 mg Zn(OA)_2_ stock solution, 5.0 mg SeTOP stock solution, and 1.1 mg HPPh_2_ (with a molar ratio of 4:1:4), in 0.50 mL of *d*_8_‐Tol. The NMR measurements are qualitatively in agreement with the hypothesis of precursor self‐assembly proposed.

**Scheme 1:**
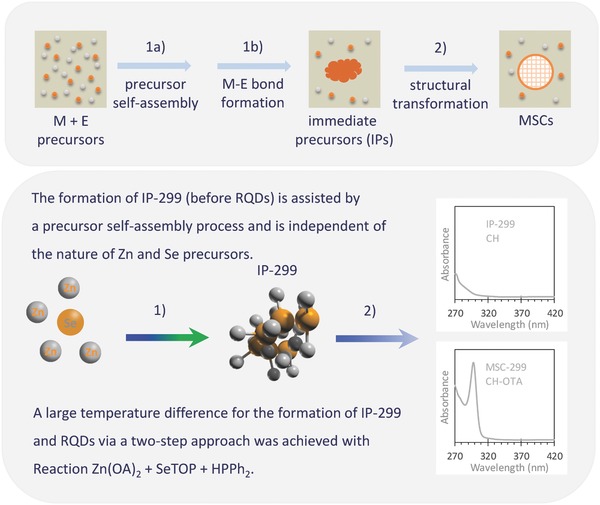
Schematic drawing of the formation pathway for colloidal semiconductor compound MSCs (top panel) starting with precursor self‐assembly followed by M—E covalent bond formation, which leads to IPs being produced. The general reactions for monomers, MSCs, and RQDs were reported based on the same P‐containing products monitored by ^31^P NMR,^[26‐29]^ while the structural transformation from IPs to MSCs was explored in detail.^[39a]^ With ZnSe as a model system (bottom panel), the formation of IP‐299 prior to RQDs, together with the detection of ZnSe MSC‐299 from reactions with various Zn and Se precursors suggests a general precursor self‐assembly process which takes place prior to the Zn—Se bond formation.

Optical absorption spectroscopy was used to explore the induction period of each of the reactions, for the presence of IP‐299 which led to the formation of ZnSe MSC‐299. **Figure** **1** and Figure S1 (Supporting Information) present the absorption spectra for the first two reactions, while **Figure** **2** and Figure S2 (Supporting Information) address Reaction (3). **Figure** **3** and Figure S3 (Supporting Information) deal with Reactions (4). Among the reactions explored, Reaction (4) Zn(OA)_2_ + SeTOP + HPPh_2_ was identified to be the most efficient for synthesizing ZnSe MSC‐299 in a single‐ensemble form, with the optimized experimental window demonstrated in **Figure** **4** and Figure S4 (Supporting Information). Furthermore, the reaction mixtures developed in the first step of the two‐step approach were studied by electrospray ionization mass spectrometry (ESI‐MS), ^1^H NMR, ^31^P—^1^H heteronuclear multiple bond correlation (HMBC) NMR, and ^1^H NMR with diffusion ordered spectroscopy (DOSY), as shown in **Figure** **5** and Figure S5 (Supporting Information). Importantly, the Reaction (4) temperature for the formation of IP‐299 could be about 120 °C lower than that for the nucleation/growth of RQDs upon optimization. The lower temperature for the formation of IP‐299 is in complete agreement with precursor self‐assembly in the first step of our two‐step approach, as displayed by **Scheme** **1** and Scheme S1 (Supporting Information). Scheme S1 (Supporting Information) indicates that monomers and IPs are different species with different formation pathways.^9^ Finally, we discuss in Note S1 (Supporting Information) the challenge of conventional characterization tools for the size and structure of the MSCs.

### 2.1 Reactions (1)and(2)

Figure 1 presents the exploration of the induction period via an optical absorption spectroscopy study for Reaction (1) Zn(OA)_2_ + SeTOP. The two precursors have been used previously to synthesize ZnSe RQDs,^52^ as summarized in Table S1 (Supporting Information). Parts a and b of Figure 1 show the evolution with temperature of the absorption of nine samples (15 µL) in 3.0 mL of cyclohexane (CH) and in a mixture of 1.0 mL CH and 2.0 mL octylamine (OTA), respectively. The nine samples were obtained at various reaction temperatures, which increased from 80 to 240 °C in the step of 20 °C. The absorption spectra collected from one sample but in the two dispersions are compared and shown in parts c–f of Figure 1, for the four 180–240 °C samples. For the remaining five samples collected from 80 to 160 °C, the similar comparisons are presented in Figure S1‐1 (Supporting Information).

For the cyclohexane dispersions (parts a, c, and d of Figure 1), no significant absorption peaks were detected above 290 nm, for the 80–200 °C samples. For the remaining two 220 and 240 °C samples (parts a, e, and f of Figure 1), ZnSe RQDs were detected which exhibited bandgap absorption peaks at ≈375 and ≈390 nm, respectively. Thus, the nucleation/growth of RQDs appeared to occur at temperatures above ≈200 °C.

For the CH–OTA mixture dispersions (parts b, d, and e of Figure 1), a sharp absorption peak at 299 nm was detected, but only for the two samples at 200 and 220 °C. This sharp absorption peak provides the information regarding the evolution of IP‐299 in these two samples, as well as the indication of the presence of MSC‐299. It is apparent that the population of MSC‐299 increased from the 200 °C sample to the 220 °C sample. On the other hand, for the 180 and 240 °C two samples (parts b, c, and f of Figure 1), no such sharp absorption peak was detected; the absence of MSC‐299 indicates that IP‐299 was also not present in the two samples. From these results, we can reasonably conclude that the formation of IP‐299 did not take place at 180 °C, while IP‐299 was not stable at a temperature as high as 240 °C due to the growth of RQDs.^39^

There is an indication that the formation of IP‐299 in the reaction of Zn(OA)_2_ + SeTOP requires a temperature as high as 200 °C. We then used a different Se precursor (SeODE) but the same Zn precursor Zn(OA)_2_ (Reaction (2) as shown by Figure S1‐2, Supporting Information).^36^, ^37^, ^57^ Interestingly, MSC‐299 was detected in the mixture of 2.0 mL CH and 1.0 mL OTA, and the formation of IP‐299 in the reaction of Zn(OA)_2_ + SeODE seemed to require a temperature as high as 180 °C, with the presence of RQDs at 220 °C.

For Reactions (1) and (2) with the two different Se precursors, MSC‐299 was detected in CH–OTA dispersions but not in CH dispersions. Such an evolution of MSC‐299 provides indirect but compelling evidence for the presence of IP‐299 which appears likely to be macromolecular in nature. Furthermore, the self‐assembly of Zn and Se precursors, which leads to IP‐299 at relatively low temperatures as compared to those of RQDs, could be a general phenomenon and independent of the nature of the two precursors used. The detection of MSC‐299 from Reactions (3)–(5) as shown below reinforces our hypothesis that the formation of IP‐299 is precursor independent and is driven by precursor self‐assembly.

To further explore the concept of the self‐assembly process which takes place prior to the formation of the Zn—Se covalent bond, we used lower Se feed concentrations for Reactions (1) and (2) as demonstrated by Figure S1‐3a,b (Supporting Information), respectively. The existence of a critical micellization concentration (CMC) for surfactants to self‐assemble in an aqueous solution is well established.^58^, ^59^ Without “micellar‐like” aggregates, there would be no IP‐299 formed, for which reason MSC‐299 would not be detected in the CH–OTA dispersion. Indeed, little MSC‐299 was observed to be present in the CH–OTA dispersion, while RQDs appeared at ≈240 °C, as shown in Figure S1‐3 (Supporting Information). It seems that the formation of “micellar‐like” aggregates requires the concentration of SeTOP and SeODE greater than CMC 30 mmol kg^−1^. In Figure S1‐4 (Supporting Information) which deals with Reaction (1) with higher Se feed concentrations of 120 and 180 mmol kg^−1^, we noticed again that MSC‐299 was detected. It is reasonable that the actual CMC value is related to the precursor reactivity in a reaction, as demonstrated by our exploration for Reactions (4) and (5).

### 2.2 Reaction (3) of Zn(OA)_2_ + SePPh_2_H

To further explore another Se precursor (toward the goal of decreasing the temperature required for the formation of IP‐299), we investigated Reaction (3) Zn(OA)_2_ + SePPh_2_H, focusing also on the induction period prior to the nucleation/growth of ZnSe RQDs.^52^ Figure 2 shows the optical absorption spectroscopy of this reaction when its temperature was increased from 40 to 200 °C in uniform steps of 20 °C. Figure 2 presents the evolution with temperature of the absorption for samples taken sequentially at the nine temperatures. Each sample (15 µL) was dispersed in 3.0 mL of cyclohexane (a) and in the mixture of 2.0 mL CH and 1.0 mL OTA (b). The absorption spectra for the four samples extracted at 40 (c), 120 (d), 160 (e), and 200 °C (f) are compared between the two dispersions, with blue and red traces for the cyclohexane and CH–OTA mixture dispersions, respectively. For the nine samples collected from 40 to 200 °C, Figure S2 (Supporting Information) shows a similar comparison of their absorption spectra.

For the cyclohexane dispersions, ZnSe RQDs were detected even in the 120 °C sample, for which a bandgap absorption peak appeared at ≈325 nm. When the temperature was increased to 200 °C, the RQDs grew in size with the bandgap absorption peak redshifting to ≈360 nm. Thus, the nucleation/growth of RQDs took place at temperatures slightly above 100 °C. For the CH–OTA mixture dispersions, MSC‐299 was detected even in the 40 °C sample. Accordingly, the formation of IP‐299 must have occurred at a similarly low temperature. A decrease in the population of IP‐299 started from 160 °C due to the growth of RQDs.^39^ An apparent coexistence of ZnSe MSC‐299 and RQDs has been observed in the 120–200 °C samples. The detection of MSC‐299 from Reaction (3) at 40 °C with RQDs at 120 °C is in agreement with our hypothesis that the formation of IP‐299 and thus MSCs is driven by precursor self‐assembly and can be independent of the nature of precursors used.

### 2.3 Reaction (4) of Zn(OA)_2_ + SeTOP + HPPh_2_

For Reactions (1) and (2), the formation of the IPs seemed to occur at a temperature only slightly lower than that of nucleation/growth of ZnSe RQDs. For Reaction (3), the temperature difference seemed to be larger. A better design of the two‐step approach for the formation of a single‐ensemble MSC‐299 (without the production of RQDs) would require the temperature difference to be even larger; furthermore, the growth period at that lower temperature should be relatively long. With such an idea in mind, we employed HPPh_2_ to Reaction (1).

HPPh_2_ has been used to improve the synthetic reproducibility and particle yield of small‐size E‐based colloidal RQDs.^24^, ^52^, ^60^, ^61^, ^62^, ^63^, ^64^, ^65^ The use of HPPh_2_ was first introduced for PbSe,^24^ the synthesis of which seemed to require high Pb(OA)_2_ to SeTOP feed molar ratios.^24^, ^61^ Somewhat later, the Se exchange from TOP to HPPh_2_, as described by Equation (7), was proposed^[25]^ and supported by ^31^P NMR,^26^ together with the identification of the coordination between Cd and TOP.^26^

(7) $$\mathrm{SeTOP}+{\mathrm{HPPh}}_{2}\Leftrightarrow \mathrm{TOP}+{\mathrm{SePPh}}_{2}H$$

This equilibrium is heavily weighted toward the left,^25^, ^26^ but high M‐to‐SeTOP feed molar ratios facilitate its shift to the right due to the coordination between M and TOP.^26^, ^27^, ^28^, ^29^ SePPh_2_H is much more reactive than SeTOP.^25^, ^26^, ^27^, ^28^, ^29^, ^52^, ^60^, ^61^, ^62^, ^63^, ^65^ From these outcomes, the use of high cation‐to‐ETOP feed molar ratios was understood to facilitate the Se exchange from TOP to HPPh_2_.^26^, ^27^, ^28^, ^29^, ^60^, ^61^, ^62^, ^63^, ^64^, ^65^

Figure 3 presents the absorption spectra for the 80 (a), 100 (b), 120 (c), 200 (d), 220 (e), and 240 °C (f) samples from Reaction (4) Zn(OA)_2_ + SeTOP + HPPh_2_. Each of the samples (15 µL) was dispersed in 3.0 mL of cyclohexane (blue traces) and in 3.0 mL of the 2CH–1OTA mixture (red traces). For the investigation of the induction period, Figure S3‐1 (Supporting Information) illustrates the evolution with temperature of the absorption of the nine samples (from 80 to 240 °C) in the two dispersions. Figure S3‐2 (Supporting Information) shows the comparison of the absorption spectra collected from each of the nine samples dispersed in the two solvents. Optical absorption spectroscopy demonstrates that the use of HPPh_2_ increased the difference between the temperatures at which IP‐299 formed and RQDs nucleated and grew.

For the cyclohexane dispersions, ZnSe RQDs were observed only for the 220 and 240 °C samples, which exhibited bandgap absorption peaks at ≈375 and ≈385 nm, respectively. For the other seven samples from 80 to 200 °C, there were no significant absorption peaks above 290 nm. It thus appears that the induction period prior to the nucleation/growth of RQDs must have occurred below 220 °C, which seems to be similar to the behavior in the reaction of Zn(OA)_2_ + SeTOP without the use of the secondary phosphine HPPh_2_.

For the CH–OTA mixture dispersions, the results were radically different, as MSC‐299 was detected in all the seven samples taken from 100 to 220 °C, with a continuous increase in the MSC population from the 100 °C sample to the 200 °C sample. Above this temperature range, the MSC‐299 population decreased for the 220 °C sample and disappeared entirely for the 240 °C sample. Thus, there was apparently little formation of IP‐299 at 80 °C (Figure 3a, red trace), and essentially no IP‐299 remained at 240 °C (Figure 3f, red trace). Upon the formation taking place at a temperature as low as 100 °C (Figure 3b, red trace), the population of IP‐299 increased up to 200 °C.

For Reaction (4) of Zn(OA)_2_ + SeTOP + HPPh_2_, there is an equilibrium for the exchange of Se between TOP and HPPh_2_,^25^, ^26^, ^27^, ^28^, ^29^ as described by Equation (7). The use of ETOP and HPPh_2_ with high cation‐to‐ETOP feed molar ratios has been demonstrated to effectively decrease the temperature required for nucleation/growth of RQDs,^60^, ^61^, ^62^, ^63^, ^64^, ^65^ as also demonstrated for ZnSe.^52^ For example, ZnSe RQDs were detected at ≈160 °C with the feed molar ratios of 4Zn(OA)_2_ + 1Se1TOP + 4HPPh_2_.^52^ For ZnSe MSC‐299, the optimum feed molar ratios were explored to be 4Zn(OA)_2_ + 1Se2.2TOP + 1HPPh_2_, where IP‐299 formed at 100 °C and RQDs at 220 °C (Figure 3). Without the use of HPPh_2_, IP‐299 developed at a higher temperature of 200 °C while the QD formation temperature remained at 220 °C (Figure 1). In this case, the temperature required for the formation of IP‐299 was quite close to that needed for the RQDs. When SePPh_2_H was used directly as the Se precursor in Reaction (3), IP‐299 evolved at the significantly lower temperature of 40 °C, while the QD formation temperature was reduced to 120 °C. These two reductions can be attributed to the fact that the available amount of SePPh_2_H in Reaction (3) was much larger than that in Reaction (4), and this amount has a strong influence on the kinetics of the formation of IP‐299 and RQDs.

Thus, in addition to the feed molar ratio of Zn to Se, the two phosphine amounts also play a key role. With optimized conditions for Reaction (4), the temperature differential between the formation of IP‐299 and RQDs was even larger. Again, the faster kinetics for the formation of IP‐299 than that for RQDs is consistent with the hypothesis of precursor self‐assembly leading to a dense‐phase reaction. Therefore, the pathways for the formation of ZnSe IP‐299 and RQDs would appear to be different.^38^, ^39^ Significantly, there is a large range of conditions that enables IP‐299 to be formed in the induction period, including the amount of HPPh_2_ used (as shown by Figure S3‐3, Supporting Information) as well as the reaction period at a constant temperature (as demonstrated by Figure 4 and Figure S4, Supporting Information).

Based on our experimental results with the two‐step approach to sole ensemble MSC‐299, Reaction (4) is the most effective and has two advantages. One is that its induction period covers a broad temperature range (below 220 °C). The other is that the formation of IP‐299 begins at temperatures as low as 100 °C. For this reason, we further explored Reaction (4) with a view to optimizing the growth of sole ensemble MSC‐299. We monitored the reaction efficiency at various discrete growth temperatures, namely, 140, 160, and 180 °C. As presented previously, each of the samples (15 µL) was dispersed in 3.0 mL of CH and of the mixture containing 2.0 mL CH and 1.0 mL OTA at room temperature. Figure S4‐1 (Supporting Information) shows the absorption spectra of these samples (from the three batches) dispersed in the two solvents. Evidently, the induction period varies inversely with the reaction temperature, with the period of ≈240, 100, and 40 min for the fixed reaction temperature of 140, 160, and 180 °C, respectively. For the CH dispersions, MSC‐299 was not detected. For the CH–OTA dispersions, MSC‐299 was generally detected (except for the 180 °C batch samples with the reaction periods longer than 100 min). ZnSe MSC‐299 exhibits little bandgap photoluminescence similar to CdTe MSCs,^38^ CdSe MSCs,^66^, ^67^, ^68^ and CdS MSCs.[[qv: 39a,c]]

Figure 4 (top) presents the absorption spectra of the 50 min sample from the 160 °C batch dispersed in the two solvents, while Figure 4 (bottom) summarizes the optical absorption spectroscopy results for these three batch samples dispersed in the CH and OTA mixture. The optimal conditions for the growth of IP‐299 would appear to be at a temperature of 160 °C for a 50 min reaction period, as shown in the top part of Figure 4. The bottom part of Figure 4 plots the absorbance strength at 299 nm of MSC‐299 in the 2CH‐1OTA dispersions versus the growth period at 140, 160, and 180 °C. At 140 °C (triangular symbols), the optical density at 299 nm increased with time up to ≈120 min. Afterward, the optical density changed little, suggesting that IP‐299 was stable (up to at least 180 min). At 160 °C (circular symbols), the optical density at 299 nm increased for 50 min and then decreased. From 30 to 50 min, the increase was slower (than that in the beginning), suggesting that this could be a practical window in which it is possible to synthesize a specific amount of IP‐299 with high synthetic reproducibility. In such an experimental window, the formation of single ensemble MSC‐299 is enabled without other‐size NCs being formed (as shown in the top part of Figure 4). At 180 °C (square symbols), the optical density at 299 nm increased in the initial 30 min and then decreased, a trend which bears a strong resemblance to the behavior at 160 °C. Even with an induction period as short as 40 min, MSC‐299 coexisted with RQDs in the temporal growth window from 50 to 80 min. Once more, formation of IP‐299 is the much readier than that of RQDs, which is in agreement with the hypothesis that the former is due to precursor self‐assembly leading to a dense‐phase reaction.

### 2.4 Reaction (4) Products Explored by MS and NMR

As shown in Figure 5 and Figure S5 (Supporting Information), we used ESI‐MS and NMR to investigate the intermediates produced in the induction period for the Zn(OA)_2_ + SeTOP + HPPh_2_ reaction. ESI‐MS has been used to study various nanoclusters,^69^, ^70^, ^71^ as well as the formation of intermediates in induction periods of colloidal semiconductor quantum dots.^38^, ^39^ Figure 5 (top) shows two ESI‐MS spectra, which were obtained using the negative‐ion mode within the *m*/*z* range from 300 to 1600 Da and which were measured for the samples taken at 40 °C/5 min and 160 °C/15 min. For this reaction, Figure S5‐1a (Supporting Information) shows the evolution with temperature of the UV–vis absorption of the samples extracted at different reaction temperatures and growth periods and then dispersed in 3.0 mL of CH and the 2CH–1OTA mixture. Figure S5‐1b (Supporting Information) shows the ESI‐MS study results for six samples taken at 40, 120, and 160 °C, with Figure S5‐1c (Supporting Information) containing the MS isotopic patterns simulated for Zn, Se, and Zn_1_Se_1_. It has been reported that the II–VI clusters detected by MS are free of surface ligands, which have low‐dissociation energy from the surface atoms and could be detached readily.^38^, ^39^, ^66^, ^72^, ^73^

For the 40 °C/5 min sample with ESI‐MS, cluster fragments (rather than the starting compound and/or cluster due to the high energy of the electrospray ionization) were detected consisting of Zn and Se isotopic peaks in the *m*/*z* range of 300–1600 Da. In the *m*/*z* range of 300–800 Da, the small cluster fragments detected have been assigned to Zn_3_Se_3_, Zn_4_Se_5_, Zn_6_Se_4_, Zn_4_Se_6_, and Zn_6_Se_5_. Figure S5‐1d (Supporting Information) contains a detailed assignment of these various peaks which are isotopic‐weighted. In the *m*/*z* range of 1000–1600 Da, two cluster peaks were detected with significant strength at 1027 and 1280 Da, which were assigned to Zn_6_Se_8_ and Zn_10_Se_8_, respectively. These two peaks were also detected in the 120 and 160 °C samples (Figure S5‐1b, Supporting Information). Clearly, the formation of Zn—Se covalent bonds took place at 40 °C; interestingly, no MSC‐299 was detected in the 40 °C sample as shown in Figure S5‐1a (Supporting Information).

For the 160 °C/15 min sample, ESI‐MS indicated that there were 12 to 21‐atom Zn*_x_*Se*_y_* cluster fragments in the *m*/*z* range of 800–1600 Da. In the *m*/*z* range of 880–1400 Da, Zn_8_Se_4_ seemed to have a strong strength, with the formula of Zn*_x_*Se_8_ (*x* = 4–11) being indicative of the other dominant cluster. The peaks with less intensity were attributed to Zn*_x_*Se_9_ (*x* = 4–9) and Zn*_x_*Se_6_ (*x* = 6–12), in addition to Zn_7_Se_5_ and Zn_8_Se_5_, as shown in Figure S5‐1e (Supporting Information). In the *m*/*z* range of 1400–1600 Da, clusters described by the general formula Zn*_x_*Se_12_ (*x* = 7–9) were detected. Figure S5‐1e (Supporting Information) shows the detailed assignment of these isotopic peaks. These peaks detected in the 160 °C samples were also present in the 120 °C sample (Figure S5‐1b, Supporting Information), for which a small amount of MSC‐299 was apparent, but only when dispersed in the CH–OTA mixture (Figure S5‐1a, Supporting Information). For the 160 °C samples taken from the reaction between 5 and 45 min, the MSC‐299 population increased considerably up to 15 min and then became stable.

On the whole, the formation of Zn—Se covalent bonds took place in the 40 °C sample; however, IP‐299 was not produced. For the 120 °C sample, there was much less IP‐299 formed than that in the four 160 °C samples. Such an observation is in agreement with the results shown by Figure 4 that the favorable temperature for the evolution of IP‐299 was 160 °C. Figure 5 (top) and Figure S5‐1 (Supporting Information) provide strong experimental evidence that in the transformation from “micellar‐like” aggregates to ZnSe IP‐299, Zn—Se covalent bonds were continuously formed in the large experimental window from 40 to 160 °C.

Figure 5 (bottom) and Figure S5‐2 (Supporting Information) present our solution NMR studies at room temperature of two toluene solutions as indicated. It has been acknowledged that the mobility of a species in solution is related to its size, with a larger species exhibiting a smaller diffusion coefficient.^8^, ^38^ The specific relationship between the mobility (*D*, diffusion coefficient) and size (*d*_H_, hydrodynamic diameter) follows the Stokes–Einstein equation

(8) $$D=k_{B}\;T/3\pi \;\eta d_{H}$$

with *k*_B_ the Boltzmann constant and η the dynamic viscosity of the liquid. For the two toluene solutions from left to right, the toluene (7.1 ppm) diffusion coefficient obtained was 10^−8.640^ and 10^−8.719^ m^2^ s^−1^, respectively. The decreased diffusion coefficient of the toluene signal in Zn(OA)_2_ + SeTOP + HPPh_2_ (right) suggests a probable increase in viscosity, which is in agreement with the precursor self‐assembly process proposed and with the formation of Zn—Se covalent bonds at 40 °C detected.

For the peaks at about 1.6 ppm in the Figure 5 (bottom) NMR spectra, they are assigned to the nearest‐neighbor proton to the P atom of SeTOP. This assignment is supported by the ^1^H NMR (left) and ^31^P—^1^H heteronuclear multiple bond correlation (HMBC) NMR (right) spectra shown in Figure S5‐2 (Supporting Information). Based on this 1.6 ppm proton, the averaged diffusion coefficient for SeTOP of the right solution was about 10^−9.349^ m^2^ s^−1^, which is smaller than that of the left solution (10^−9.106^ m^2^ s^−1^). Upon the approximation of viscosity with the toluene signal, the 1.6 ppm proton in Zn(OA)_2_ + SeTOP + HPPh_2_ (right) is estimated to be associated with species about (10^−9.106^ × 10^−8.719^)/(10^−9.349^ × 10^−8.640^) = 1.5 times in size, as compared to that in SeTOP (left). Thus, the DOSY measurements seem to be qualitatively consistent with the hypothesis of precursor self‐assembly proposed.

### 2.5 On Precursor Self‐Assembly Explored by the Two‐Step Approach

Scheme 1 and Scheme S1 (Supporting Information) present our hypothesis of the formation pathway of colloidal semiconductor MSCs. The pathway starts with precursor self‐assembly, which leads to a dense phase reaction to IPs with M—E covalent bond formation at a relatively low temperature as compared to that of RQDs. With ZnSe as a model systems, Reactions (1)–(5) with the different Zn and Se precursors all produced MSC‐299 (but with varying efficacy). Figure S6‐1,2 (Supporting Information), respectively, shows the absorption spectra for Reactions (5) and (6) with Zn(OAc)_2_/OLA as a Zn precursor. With Reaction (5), IP‐299 formed faster than RQDs did.

To obtain a product with high purity, yield, and reproducibility from a crystallization process, a fundamental understanding of the processes is of help. To comprehend more fully the two‐step approach employed to achieve ZnSe MSC‐299, we will briefly review the formation of IPs and RQDs reported for the two other relevant systems using a two‐step approach.^38^, ^39^ It was reported that the formation of CdTe IP‐371 was at 135 °C/10 min^38^ and CdS IP‐311 at 180 °C/15 min,^39^ while CdTe RQDs at 135 °C/30 min and CdS RQDs at 200 °C/15 min. When Cd and E precursors were mixed (where E = Te^38^ and Se^39^ and S^[39a–c]^), the noncovalent interaction between them resulted in the formation of “micellar‐like” aggregates prior to the formation of Cd—E covalent bonds. As the reaction progressed, Cd—E covalent bonds started to form inside the self‐assembled aggregates, leading eventually to the formation of the IPs. It was demonstrated that the whole process of the IP formation could be controlled to take place only in the induction period (as illustrated in Scheme S1, Supporting Information). By the same token,^38^, ^39^ the CdTe, CdSe, and CdS IPs occur faster than the corresponding RQDs do, and the difference in the kinetics is attributable to precursor self‐assembly leading to dense‐phase reactions for the former.

We anticipate that the present understanding of the formation of ZnSe IP‐299 via the self‐assembly of the various Zn and Se precursors will help comprehend molecular association,^1^, ^2^, ^3^, ^4^, ^5^, ^6^, ^7^, ^8^ the concept of which has been explored to advance the science of crystallization (such as for crystalline organic materials due to increasing complexity from industries).^9^, ^10^, ^11^, ^12^, ^13^, ^14^, ^15^, ^16^, ^17^, ^18^ While the interpretation of the pathway to the IP formation from the starting precursors via self‐assembly is in its infancy, molecular simulation could be able to connect the thermodynamic nature of the molecular self‐association taking place during the initial stage of crystallization, and to provide the composition information regarding the resulting IP.

## 3 Conclusions

We have demonstrated that precursor self‐assembly can be a general pathway for the formation of colloidal semiconductor MSCs, based on a study of the ZnSe model system (Scheme 1 and Scheme S1, Supporting Information). With a two‐step approach, MSC‐299 (displaying a single sharp optical absorption peak at 299 nm) has been obtained from reactions with different Zn and Se precursors. The formation of IP‐299 occurs in the first step (prior to that of RQDs); the underlying cause is attributed to a dense phase reaction for the former driven by precursor self‐assembly. The precursor self‐assembly is followed by covalent bond formation, leading to the formation of the IP. IP‐299 is optically transparent in cyclohexane (at wavelengths longer than 299 nm) and transforms into MSC‐299 in the second step when a primary amine is present. The present study presents the synthesis of the first colloidal semiconductor ZnSe MSC‐299, with a fundamental understanding of the formulation for M_2_E*_n_* MSCs that have evolved in a single‐ensemble form without the coproduction of RQDs. The hypothesis that the formation of MSCs begins with general self‐assembly of M and E precursors enriches the Yu pathway that has been proposed for the induction period of colloidal semiconductor compound NCs (Scheme S1, Supporting Information).^26^, ^27^, ^28^, ^29^, ^38^, ^39^ Efforts are needed to narrow the knowledge gap on the structure–property relationship of ZnSe MSC‐299 and ZnSe IP‐299, together with the role of the primary amine; more discussions can be found in Note S1 (Supporting Information). The present study brings more depth to our understanding of the induction period, which occurs prior to nucleation/growth of RQDs (Scheme S1, Supporting Information). The self‐assembly pathway hypothesized in the present study provides insight into the high‐concentration‐based synthesis of CdS MSCs reported recently.^40^ In addition to the other systems,^1^, ^2^, ^3^, ^4^, ^5^, ^6^ self‐assembly processes have been reported recently for the formation of polymeric vesicles (via the control of two monomer feed amounts) and polyoxometalate (POM).^7^, ^8^ We believe that the self‐assembly process reported embraces the advances of cluster science and the science of crystallization, while providing impetus for the development of desirable nucleation theories (with the concept of degree of supersaturation modified) after classical nucleation theory (CNT) and those nonclassical alternatives (Scheme S2, Supporting Information).^2^, ^9^, ^10^, ^11^, ^12^, ^13^, ^14^, ^15^, ^16^, ^17^, ^18^, ^36^, ^37^, ^38^, ^39^, ^40^, ^43^, ^44^, ^45^, ^60^, ^66^, ^67^, ^68^, ^72^, ^73^, ^74^, ^75^

## Conflict of Interest

The authors declare no conflict of interest.

## Supporting information

Supplementary
