# Supplementary material for: Precursor Self‐Assembly Identified as a General Pathway for Colloidal Semiconductor Magic‐Size Clusters
Source: Adv Sci (Weinh). 2018 Oct 23;5(12):1800632. doi: 10.1002/advs.201800632 (PMC6299716; doi:10.1002/advs.201800632)
Supplement: Supplementary file 1 — Supplementary [file ADVS-5-1800632-s001.pdf]

Copyright WILEY-VCH Verlag GmbH & Co. KGaA, 69469 Weinheim, Germany, 2018.

ADVANCED SCIENCE
Open Access

Supporting Information

for *Adv. Sci.,* DOI: 10.1002/advs.201800632

Precursor Self-Assembly Identified as a General Pathway for Colloidal Semiconductor Magic-Size Clusters

Linxi Wang, Juan Hui, Junbin Tang, Nelson Rowell, Baowei Zhang, Tingting Zhu, Meng Zhang, Xiaoyu Hao, Hongsong Fan, Jianrong Zeng, Shuo Han, and Kui Yu**

Supporting Information

Precursor Self-Assembly Identified as a General Pathway for Colloidal Semiconductor Magic-Size Clusters

Linxi Wang[1], Juan Hui[1], Junbin Tang[1], Nelson Rowell[2], Baowei Zhang[1], Tingting Zhu[1], Meng Zhang[1], Xiaoyu Hao[1], Hongsong Fan[3], Jianrong Zeng[4], Shuo Han[1], Kui Yu[1,3,5]

[1]Institute of Atomic and Molecular Physics, Sichuan University, 610065, P. R. China

[2]National Research Council of Canada, Ottawa, Ontario K1A 0R6, Canada

[3]Engineering Research Center in Biomaterials, Sichuan University, 610065, P. R. China

[4]Shanghai Synchrotron Radiation Facility, Shanghai Institute of Applied Physics, Chinese Academy of Sciences, Shanghai, 201204, P. R. China

[5]School of Chemical Engineering, Sichuan University, 610065, P. R. China

Table of contents

Experimental		S2
Table S1a.	Summary of literature reports on the synthesis of ZnSe clusters	S6
Table S1b.	Summary of literature reports on the synthesis of ZnSe RQDs	S7
Figure S1-1a.	Reaction (1) [SeTOP] = 60 mmol/Kg absorption comparison	S8
Figure S1-1b.	Absorption spectra of oleic acid (OA)	S9
Figure S1-2.	Reaction (2) [SeODE] = 60 mmol/Kg absorption spectra	S10
Figure S1-3a.	Reaction (1) [SeTOP] = 30 mmol/Kg absorption spectra	S12
Figure S1-3b.	Reaction (2) [SeODE] = 15 and 30 mmol/Kg absorption spectra	S13
Figure S1-4.	Reaction (1) [SeTOP] = 120 and 180 mmol/Kg absorption spectra	S14
Figure S2.	Reaction (3) [Se] = 60 mmol/Kg absorption comparison	S15
Figure S3-1.	Reaction (4) [Se] = 60 mmol/Kg absorption spectra	S16
Figure S3-2.	Reaction (4) absorption comparison	S17
Figure S3-3.	Reaction (4) $4Zn(OA)_2$ + 1SeTOP + $xHPPh_2$ absorption spectra	S18
Figure S3-4.	Reaction (4) [Se] = 30 mmol/Kg absorption spectra	S19
Figure S4-1a.	Reaction (4) 60 mmol/Kg at 140 °C absorption spectra	S20
Figure S4-1b.	Reaction (4) 60 mmol/Kg at 160 °C absorption spectra	S21

Figure S4-1c. Reaction (4) 60 mmol/Kg at 180 °C absorption spectra S22
Figure S4-2a. Reaction (4) 30 mmol/Kg at 140 °C absorption c spectra S23
Figure S4-2b. Reaction (4) 30 mmol/Kg at 180 °C absorption spectra S24

Figure S5-1a. Reaction (4) batch for MS absorption spectra S25
Figure S5-1b. MS of the five samples from Fig. S5-1a batch S26
Figure S5-1c. Simulated isotope distribution for Zn, Se, and Zn_1Se_1 S26
Figure S5-1d. MS cluster fragments for the Fig. S5-1a batch 40 °C/5 min sample S27
Figure S5-1e. MS cluster fragments for the Fig. S5-1a batch 160 °C/15 min sample S28
Figure S5-2. NMR study of precursor self-assembly of Reaction (4) S29

Figure S6-1a. Reaction (5) with $Zn(OAc)_2$/OLA [Se] = 60 mmol/Kg absorption S30
Figure S6-1b. Reaction (5) with $Zn(OAc)_2$/OLA [Se] = 30 mmol/Kg absorption S31
Figure S6-2a. Reaction (6) with $Zn(OAc)_2$/OLA [Se] = 30 and 60 mmol/Kg S32
Figure S6-2b. Reaction (6) with $Zn(OAc)_2$/OLA [Se] = 120 and 180 mmol/Kg S33

Figure S6-3a. The use of BTA instead of OTA S34
Figure S6-3b. The use of di-BTA instead of BTA S35
Figure S6-4a NMR of a mixture of a Zn precursor and a primary amine S36
Figure S6-4b NMR of mixtures of a Zn precursor and secondary/tertiary amines S37
Figure S6-5a. TEM and XRD study S38
Figure S6-5b. Stability of MSC-299 purified once S39
Figure S6-6. Stability of unpurified MSC-299 in the mixtures of CH and OTA S40

Scheme S1 Schematic drawing of the exploration of the induction period S41
Scheme S2. Schematic drawing to compare with the two-step nucleation S42

Figure S7 CdSe MSC-415 from two Cd precursors reactions with SeTOP S43

Note S1 On the second step and conventional characterization tools S44

EXPERIMENTAL

Zinc selenide (ZnSe) was used as a model system. The resulting clusters are denoted as MSC-299, based on the wavelength in nanometers of their absorption peak. ZnSe MSC-299 has not been reported before, to the best of our knowledge.

1. Chemicals and Stock Solution Preparation

Commercial chemicals, including zinc oxide (ZnO, 99.0%, Chengdu Kelong Chemical), zinc acetate ($Zn(OAc)_2$, 99.99%, Aldrich), selenium powder (Se, 99.99%, Alfa Aesar), tri-*n*-octylphosphine (TOP, 90.0%, Aldrich), diphenylphosphine (PPh_2H, 98.0%, J&K), oleic acid (OA, 90%, Aldrich), 1-octadecene (ODE, 90.0%, Aldrich), octylamine (OTA, 99.0%, Aldrich), and cyclohexane (CH, 99.5%, Taiyang) were used as received. Toluene (Tol, 99.5%, Chengdu Kelong Chemical) was distilled and ~ 250 mL of distilled toluene was further dried with ~ 20 g of KOH (99.5%, Chengdu Kelong Chemical).

To prepare a $Zn(OA)_2$ stock solution (with the total weight of 9.221 g and the concentration of $Zn(OA)_2$ of 0.545 mmol/mL), ZnO (0.492 g, 6.04 mmol), OA (3.729 g, 13.20 mmol) and ODE (5.000 g) were added into a 50 mL three-necked reaction flask at room temperature and the flask was evacuated for ~ 30 min, until no bubbles were observed. Under a nitrogen (N_2) atmosphere, the mixture was then heated up to 120 °C, and it was placed under vacuum at this temperature for two hours. Afterwards, under a nitrogen (N_2) atmosphere, the mixture was heated up to 290 °C to form a clear solution, which was then cooled down to 120 °C. This mixture was subjected to vacuum for two hours. The stock solution was stored in a glovebox at room temperature, which was also how the stock solutions described below were stored.

To prepare a SeTOP stock solution, Se powder (0.328 g, 4.15 mmol), TOP (3.390 g, 9.15 mmol) and ODE (0.437 g), were put in a 25 mL three-necked reaction flask at room temperature. The total weight of the reaction mixture was ~4.150 g with the Se concentration of 1 mmol/g). The mixture was stirred at 40 °C for 20 min to achieve a clear solution under vacuum.

To prepare $SePPh_2H$, Se powder (0.024 g, 0.30 mmol) and $HPPh_2$ (52 μL, 0.30 mmol) were mixed and then heated at ~90 °C for 1 hr in the glove box until all the Se powder disappeared completely. The $SeHPPh_2$ solid was opaque and white (a little yellow) in

appearance at room temperature.

2. Synthesis of ZnSe MSC-299 via a Two-step Approach

For the first step of a typical reaction of $Zn(OA)_2$ + SeTOP, a mixture of the $Zn(OA)_2$ stock solution (2.2 mL, 1.20 mmol) and ODE (3.3 mL) was placed in a 50 mL three-necked flask. The flask was evacuated and then backfilled with N_2 gas; this procedure was repeated three times. Then, the mixture was heated to 80 °C under N_2 and vacuumed for 30 min at this temperature. Next, under a N_2 atmosphere, the mixture was heated up to 120 °C and then was held under vacuum for 2 hrs, followed by cooling in a N_2 atmosphere to 80 °C. A mixture of SeTOP (330 μL, 0.30 mmol) and ODE (0.4 mL) was added to achieve a feed molar ratio of 4 to 1 of $Zn(OA)_2$ to SeTOP, a Se concentration of either 60 or 30 mmol/kg, and a total weight of the reaction mixture of 5.000 g. The reaction flask was evacuated and then backfilled with N_2 gas three times. Under a N_2 atmosphere, the reaction mixture was kept at 80 °C for 15 min, and then heated to 100, 120, 140, 160, 180, 200, 220, and 240 °C. At each temperature after 15 min, a sample was removed from the reaction mixture.

For the first step of the reaction of $Zn(OA)_2$ + SeTOP + $HPPh_2$, a mixture of the $Zn(OA)_2$ (2.2 mL, 1.20 mmol) stock solution and ODE (3.3 mL) was prepared at 80 °C as described above in a 50 mL three-necked flask. Under a N_2 atmosphere, the Se-containing solution, which consisted of SeTOP (330 μL, 0.30 mmol), $HPPh_2$ (52 μL, 0.30 mmol), and ODE (0.4 mL) and which had been pre-mixed in a glove box, was added. The feed molar ratio of $Zn(OA)_2$ to SeTOP to $HPPh_2$ was 4 to 1 to 1, with a Se concentration of 60 mmol/kg, and a total weight of the reaction mixture of 5.000 g. Nine samples were taken from 80 °C/15 min to 240 °C/15 min with an interval of 20 °C, in a similar fashion to that described above for the reaction of $Zn(OA)_2$ + SeTOP.

For the first step of a typical reaction of $Zn(OA)_2$ + $SePPh_2H$, we prepared a mixture of the $Zn(OA)_2$ (2.2 mL, 1.20 mmol) stock solution and ODE (3.4 mL) as described above, but in this case it was kept at 40 °C. Under a N_2 atmosphere, a mixture of $SePPh_2H$ (52 μL, 0.30 mmol) and ODE (0.5 mL), which had been prepared in the glove box, was added. The feed molar ratio of $Zn(OA)_2$ to $SePPh_2H$ was 4 to 1, with a Se concentration of 60 mmol/kg and a total weight of the reaction mixture of 5.000 g. Under a N_2 atmosphere, samples were taken each 20 °C from 40 °C/15 min to 220 °C/15 min, as described for the above two reactions.

For the second step of our approach, it involves the nucleation of ZnSe MSC-299 at room temperature. A certain amount (such as 15 μL) of the reaction mixture was taken from one of the three reactions described above. Such a sample, which could have contained IP-299, was then dispersed in 3.0 mL of cyclohexane (CH) and in a mixture of 1.0 mL of CH and 2.0 mL of OTA (Figures 1 and S1-1) or 2.0 mL of CH and 1.0 mL of OTA (other figures). The presence and evolution of MSC-299 in the latter dispersion was monitored mainly by *in situ* UV-vis absorption spectroscopy.

3. Characterization

The UV-vis absorption spectra were collected between 270 nm and 500 nm with a Hitachi UH4150 spectrometer using intervals of 0.5 nm or 1 nm. The quartz cuvettes (3.5 mL standard QS cells with the light path of 10 mm) were purchased from Hellma Analytics. Background measurements were performed with cyclohexane.

Electrospray ionization mass spectrometry (ESI-MS) was performed in the negative ion mode on Agilent 6210A HPLC-TOF/MS (at Chengdu Branch, Chinese Academy of Sciences). Data analysis and instrument operation were performed with Agilent's "Mass Hunter" software. The first-step samples used were stored in dried toluene with a volume ratio of 1 to 1. For MS characterization, 1 μL of the stored sample in toluene was further dispersed in 1.0 mL of toluene, with acetonitrile as the mobile phase.

Nuclear magnetic resonance (NMR) was carried out. The ^{1}H NMR measurements were taken using an Agilent 400-MR DD2 (Figure S5-2 and Figure S6-4). The ^{1}H - ^{31}P two-dimensional NMR (heteronuclear multiple bond correlation spectroscopy (HMBC)) and ^{1}H NMR with diffusion ordered spectroscopy (DOSY) spectra were collected using a Bruker Avance III (400MHz). The ^{1}H NMR (Figure S5-2 and Figure S6-4) was performed with 0.03% tetramethylsilane (TMS) in d_8- toluene (99.5%, Acros) as an internal standard. The HMBC and DOSY spectra were taken both in d_8-toluene (99.5%, CIL). DOSY-NMR is a powerful technique to distinguish the size of colloidal nanocrystals (NCs), with the detailed conditions in corresponding figure caption and provided elsewhere.[1,2]

Transmission electron microscopy (TEM) and powder X-ray diffraction (XRD) were performed with the detailed conditions provided in corresponding figure caption (Figure S6-5).

[1] K. Yu, J. Ouyang, M. B. Zaman, D. Johnston, F. J. Yan, G. Li, C. I. Ratcliffe, D. M. Leek, X. Wu, J. Stupak, Z. Jakubek, D. Whitfield, *J. Phys. Chem. C* **2009**, *113*, 3390.
[2] M. Liu, K. Wang, L. Wang, S. Han, H. Fan, N. Rowell, J. A. Ripmeester, R. Renoud, F. Bian, J. Zeng, K. Yu, *Nat. Commun.* **2017**, *8*, 15467.

Table S1a. Summary of the literature reports on the synthesis of colloidal ZnSe NCs exhibiting relatively sharp and/or persistent optical absorption peaks.

Refs.	Zn precursors	Se precursors	nm (absorption peaks)<br>Comments
[44]	$ZnCl_2$<br>OTA solution	octylammonium-<br>selenocarbamate	279/289 (doublet)<br>reaction T 20 °C
[45]	$Zn(OAc)_2$<br>BTA solution	selenourea<br>BTA solution	279/289 (doublet)<br>328/347 (doublet)<br>reaction T 70 or 100 °C
[46]	$Zn(NA)_2$	H_2Se<br>OTA/OLA solution	280/291, 328/347 (doublet)<br>heating up 20 – 230 °C<br>injection/growth T 110, 180, 220 °C
[47]	$Zn(NA)_2$	SeTOP	328/346 (doublet)<br>reaction T 170 °C
[48]	$Zn(OAc)_2$<br>ODA solution	selenourea<br>ODA-DMF solution	328/347 (doublet)<br>hot injection T 140 °C<br>growth T 70 °C

temperature (T);
zinc nonanoate ($Zn(NA)_2$), zinc acetate ($Zn(OAc)_2$);
tri-*n*-octylphosphine selenide (SeTOP);
octylamine (OTA), oleylamine (OLA), butylamine (BTA), octadecylamine (ODA), dimethylformamide (DMF).

It is noteworthy that Ref 46 commented on NCs exhibiting one absorption peak at 319 nm, which were reported by *J. Chem. Phys*. **1986**, *85*, 2237 to have a secondary excitonic peak. In summary, in these published work, very reactive Se precursors, namely selenocarbamate,[44] H_2Se,[46] or selenourea,[45,48] were used to synthesize the ZnSe NCs exhibiting the sharp "doublets", with reaction media containing a primary amine, such as oleylamine (OLA, $C_{18}H_{35}NH_2$),[46] *n*-octylamine (OTA, $C_8H_{17}NH_2$),[44,46] and *n*-butylamine (BTA, $C_4H_9NH_2$).[45]

As a side note, NCs, which exhibit optical absorption doublets, have been interpreted as 0-dimension (Ref 46) or 1-dimension (Refs 46 and 48) or 2-dimension (Refs 44 and 45). The recent study (Ref 37) may provide some answers.

Table S1b. Summary of the literature reports on the synthesis of colloidal ZnSe RQDs.

Refs.	Zn precursors	Se precursors	nm (absorption peaks)<br>Comments
[46]	$Zn(NA)_2$	H_2Se<br>OTA/OLA solution	365 – 395<br>injection/growth T 220 or 230 °C
[49]	$ZnEt_2$	SeTOP	360 – 430<br>hot injection T 310 °C<br>growth T 270 °C
[50]	$ZnSt_2$<br>ODA	SeTBP	365 – 420<br>hot injection T 330 °C<br>growth T 310 °C
[51]	$ZnSt_2$<br>ODE	SeTOP	380 – 430<br>Injection/growth T 250 or 300 °C
[52]	$Zn(OA)_2$	SeTOP,<br>SeTOP + $HPPh_2$<br>$SePPh_2H$	315 – 400<br>heating up 80 – 280 °C
[53]	$ZnSt_2$<br>ODE	selenourea<br>OLA solution	~380<br>hot injection T 250 °C<br>growth T 220 °C
[54]	$ZnSt_2$<br>OLA + ODE	Se-ODE	360 – 440 (multi injection)<br>Growth T 270 °C
[55]	$ZnSt_2$<br>ODE	Se<br>ODE	361 – 405<br>growth T 290 °C
[56]	$Zn(OA)_2$<br>OA, ODE	Se<br>ODE	360 – 398<br>growth T 270 °C

temperature (T);
zinc nonanoate ($Zn(NA)_2$), diethylzinc ($ZnEt_2$), zinc stearate ($ZnSt_2$), zinc oleate ($Zn(OA)_2$);

tri-*n*-octylphosphine selenide (SeTOP), tri-*n*-butylphosphine selenide (SeTBP), diphenylphosphine selenide ($SePPh_2H$);
octadecylamine (ODA), octadecene (ODE), oleylamine (OLA).

In summary, Tri-*n*-octylphosphine selenide (SeTOP, $Se{=}P(C_8H_{17})_3$) or tri-*n*-butylphosphine selenide (SeTBP, $Se{=}P(C_4H_9)_3$) was employed for the production of ZnSe RQDs,[49-52] with the reaction medium usually of 1-octadecene (ODE) not containing a primary amine and with zinc stearate ($Zn(St)_2$, $Zn(OOCC_{17}H_{35})_2$)[50,51,53-55] or zinc oleate ($Zn(OA)_2$, $Zn(OOCC_{17}H_{33})_2$)[52,56] as a Zn precursor.

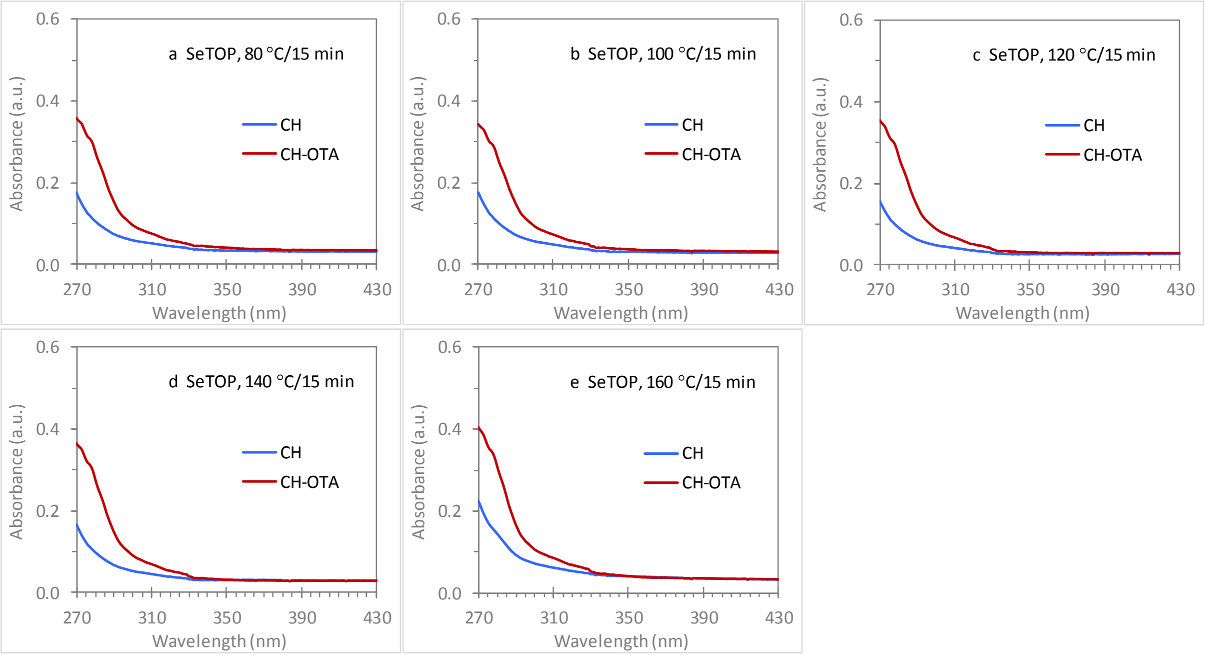

Figure S1-1a. Comparison of the absorption spectra collected from the same product (15 μL) as the Figure 1 batch, when dispersed in CH (3.0 mL, blue traces) and in the CH - OTA mixture (1.0 mL CH + 2.0 mL OTA, red traces). The growth temperature of the five samples was 80 °C (a), 100 °C (b), 120 °C (c), 140 °C (d), and160 °C (e) (as indicated). The minor peaks detected at ~279 nm were attributable to oleic acid (OA) as shown by Figure S1-1b.

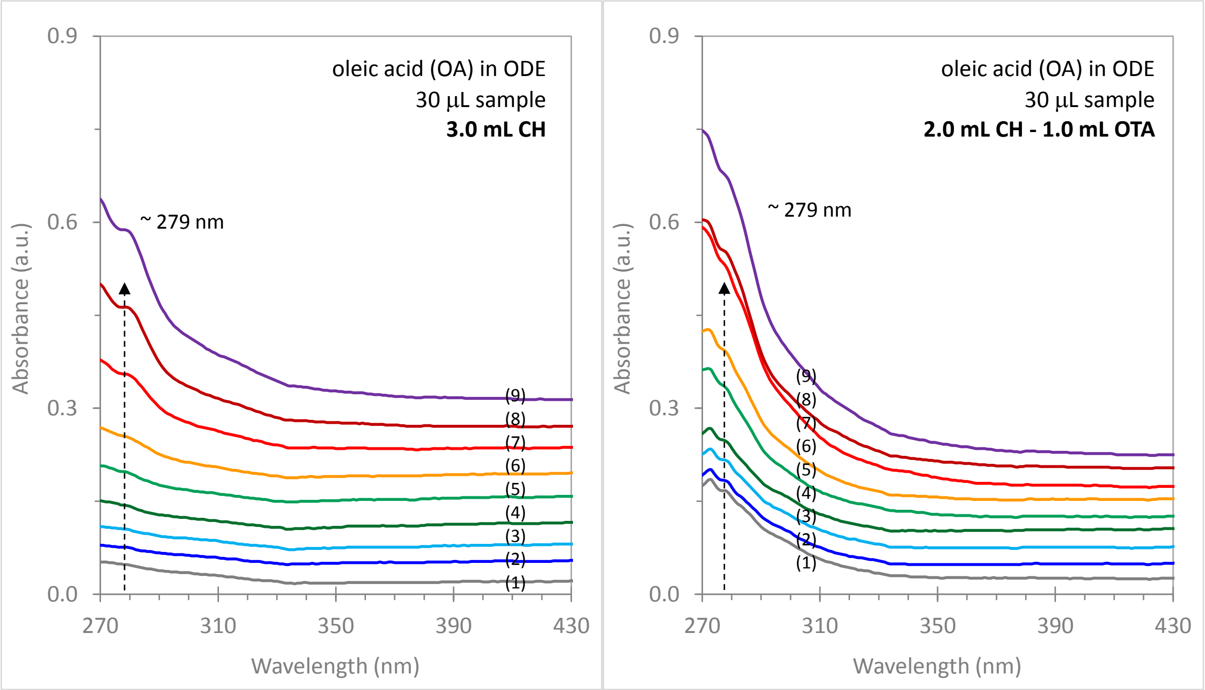

Figure S1-1b. Determination as to whether or not oleic acid (OA) caused the relatively minor peak at ~ 279 nm shown in Figures 1 and S1-1a. The same amount of OA as was used for the reaction associated with Figure 1 was placed in ODE (for a total weight of 5.000 g) and subjected to the same temperature increase. For the displayed absorption spectra, the samples were taken out after 15 min at the following temperatures (1) 80 °C, (2) 100 °C, (3) 120 °C, (4) 140 °C, (5) 160 °C, (6) 180 °C, (7) 200 °C, (8) 220 °C, and (9) 240 °C.

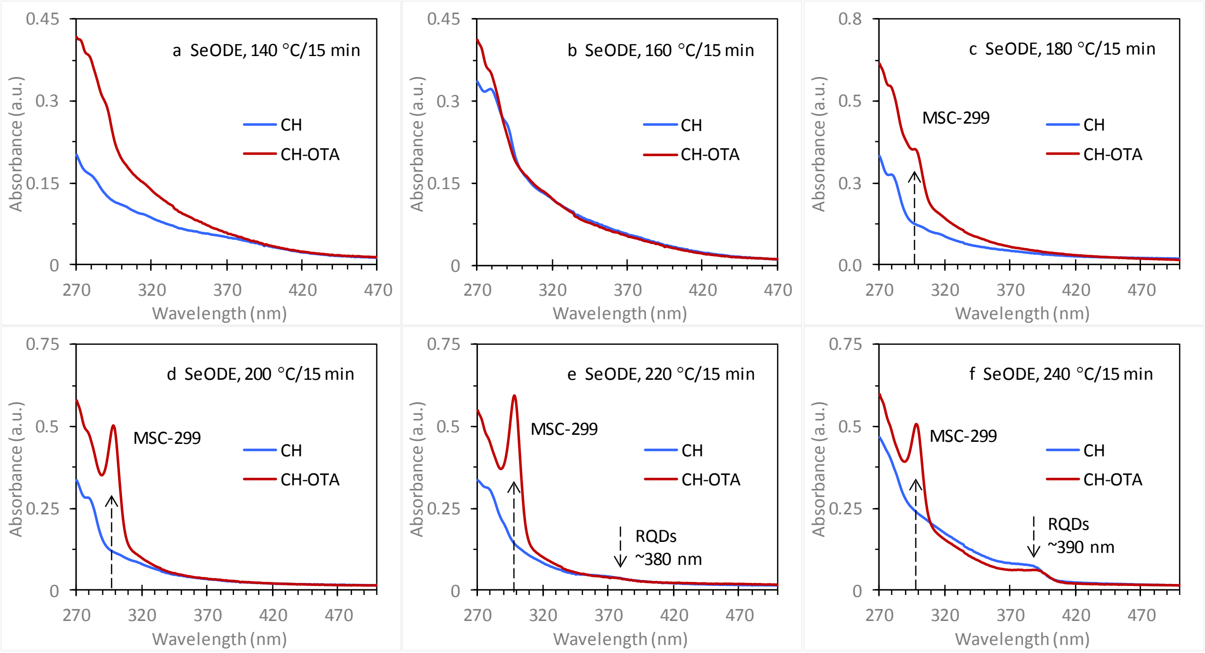

Figure S1-2. Optical absorption spectroscopy of Reaction (2) of $Zn(OA)_2$ + SeODE with a Se concentration of 60 mmol/kg. The present synthesis is similar to that of Reaction 1. For the Se precursor SeODE, Se powder (5.00 mmol) and ODE (50 mL) were added in a 100 mL three-neck flask at room temperature and evacuated for ~30 min. Under a nitrogen atmosphere, the mixture was then heated up to 180 °C and kept at this temperature for ~5 hrs to achieve a light-brown-color solution. The Zn and Se precursors were mixed at 120 °C with a feed molar ratio of 2 to 1. Samples were taken 15 min after the temperature reached (a) 140 °C, (b) 160 °C, (c) 180 °C, (d) 200 °C, (e) 220 °C, and (f) 240 °C. The absorption spectra are compared for the same sample (10 μL) dispersed in the 3.0 mL of CH (blue traces) and of the mixture (2.0 mL CH + 1.0 mL OTA, red traces). The induction period was below 220 °C. ZnSe MSC-299 was detected in the c to f samples dispersed in the CH - OTA mixture, but not when dispersed in CH. By a side note, various clusters with magic numbers of atoms have been reported, and they embrace superb stability which has been attributed to their unique structures.[1-5] For example, C_{60} and C_{70} are acknowledged to be the two most stable clusters of fullerenes, consisting of the magic carbon number of 60 and 70, respectively.[1,2] Also, metal sodium clusters containing the magic atom number 8, 20, 40 or 58 are more abundant than other-number clusters.[4] Au_{55} consisting of the magic number of 55 gold atoms with a closed-shell structure exhibits a supreme resistance to oxidation.[5] For the

present study, that the final product is of the “magic-size” type is supported by reports in the literature.[6-10] For semiconductor MSCs, experimental evidence for their presence has been based on optical measurements in which sharp absorption peaks are present at persistent positions.[6-8] The full width at half maximum (FWHM) of absorption or emission peaks of one MSC ensemble is similar to that of a single quantum dot (QD) and smaller than that of a conventional QD ensemble.[9,10]

[1] H. W. Kroto, J. R. Heath, S. C. O’Brien, R. F. Curl, R. E. Smalley, *Nature* **1985**, *318*, 162.
[2] H. W. Kroto, *Nature* **1987**, *329*, 529.
[3] A. W., J. Castleman, K. H. J. Bowen, *J. Phys. Chem. C* **1996**, *110*, 12911.
[4] M. Brack, *Sci. Am.* **1997**, *277*, 50.
[5] H. G. Boyen, G. Kästle, F. Weigl, B. Koslowski, C. Dietrich, P. Ziemann, J. P. Spatz, S. Riethmüller, C. Hartmann, M. Möller, G. Schmid, M. G. Garnier, P. Oelhafen, *Science* **2002**, *297*, 1533.
[6] A. Fojtik, H. Weller, U. Koch, A. Henglein, *Ber. Bunsenges. Phys. Chem.* **1984**, *88*, 969.
[7] V. Ptatschek, T. Schmidt, M. Lerch, G. Mu¨ller, L. Spanhel, A. Emmerling, J. Fricke, A. H. Foitzik, E. Langer, *Phys. Chem.* **1998**, *102*, 85.
[8] Z. A. Peng, X. Peng, *J. Am. Chem. Soc.* **2002**, *124*, 3343.
[9] S. A. Empedocles, R. Neuhauser, K. Shimizu, M. G. Bawendi, *Adv. Mater.* **1999**, *11*, 1243.
[10] J. Cui, A. P. Beyler, L. F. Marshall, O. Chen, D. K. Harris, D. D, Wanger, X. Brokmann, M. G. Bawendi, *Nat. Chem.* **2013**, *5*, 602.

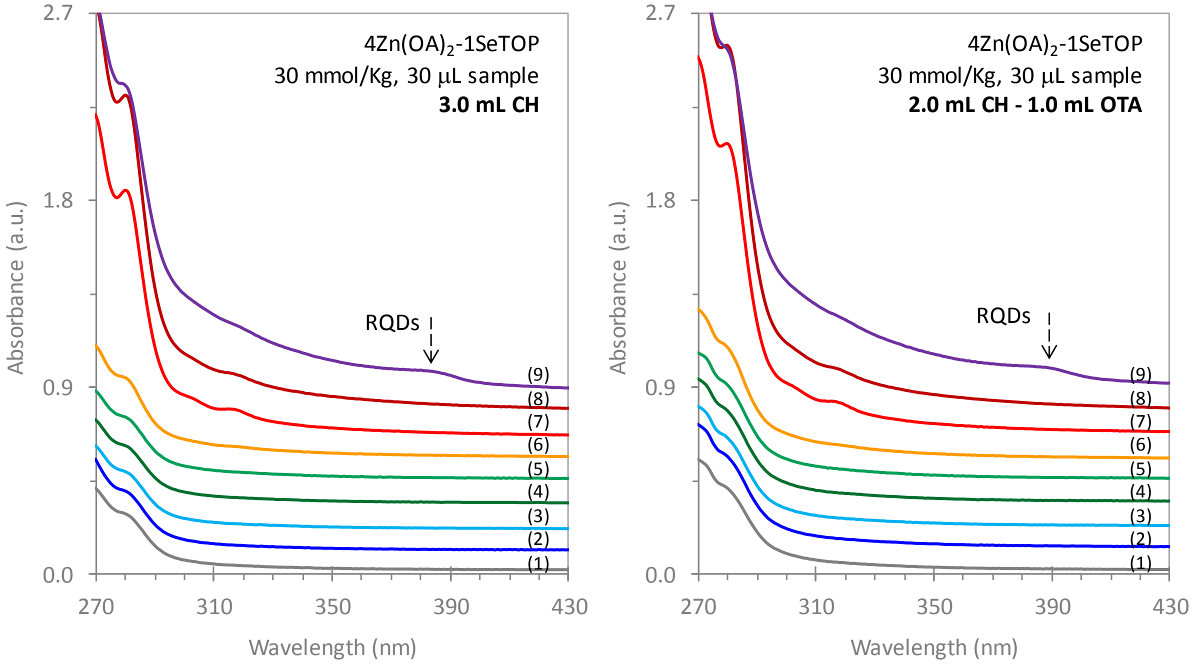

Figure S1-3a. The evolution with temperature of the absorption of the as-prepared reaction product (30 μL) dispersed in cyclohexane (CH, 3.0 mL left) and in the mixture of CH (2.0 mL) and OTA (1.0 mL) (right), from the reaction of $Zn(OA)_2$ + SeTOP. The reaction had the feed molar ratio of 4Zn to 1Se and a Se concentration of 30 mmol/Kg, with a total weight of the reaction mixture of 5.000 g. The Zn and Se precursors were mixed at 80 °C and samples were extracted after 15 min at (1) 80 °C, (2) 100 °C, (3) 120 °C, (4) 140 °C, (5) 160 °C, (6) 180 °C, (7) 200 °C, (8) 220 °C, and (9) 240 °C.

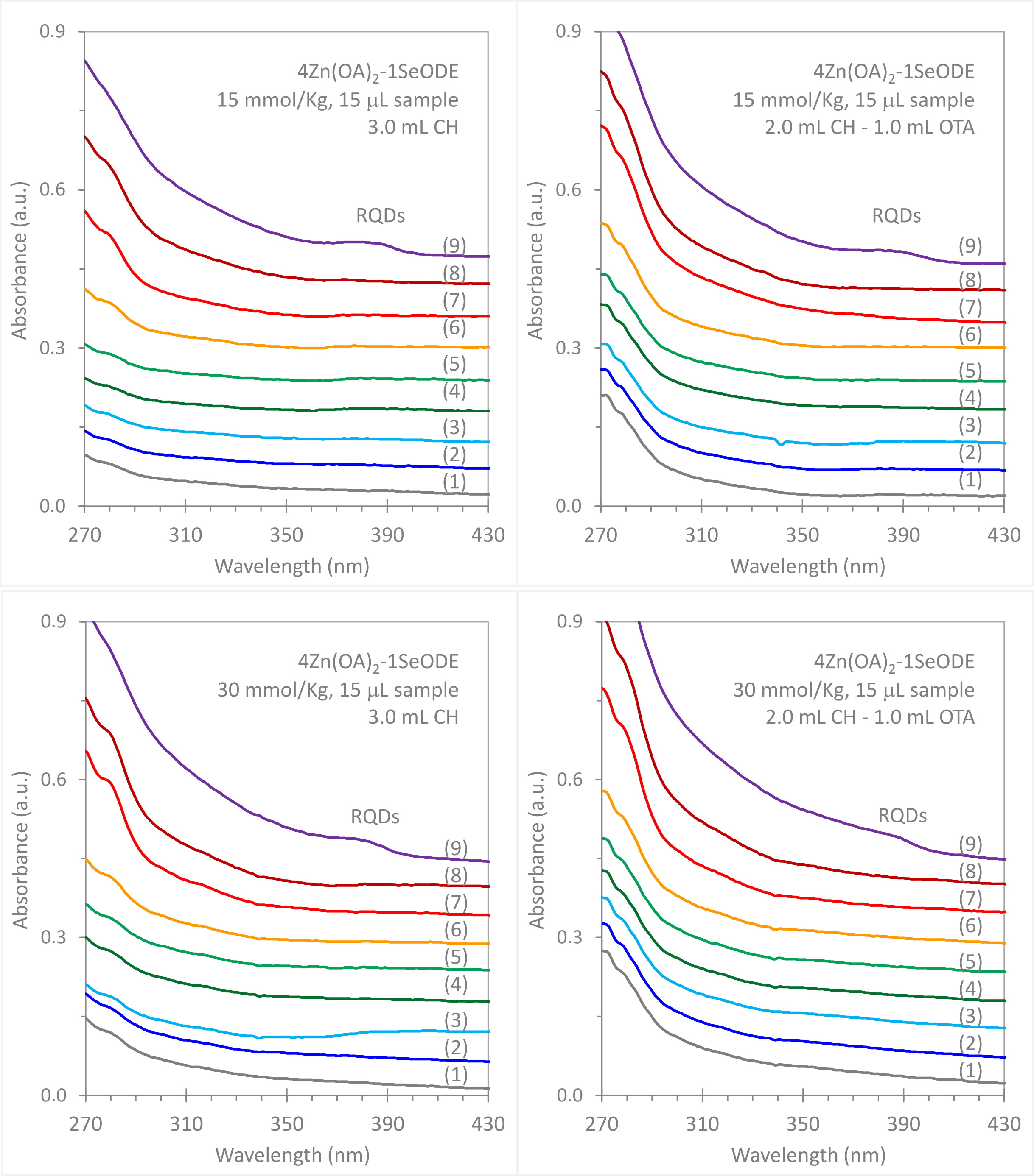

Figure S1-3b. Optical absorption spectroscopy study of the reaction of $Zn(OA)_2$ + SeODE similar to Figure S1-2 but with the Se concentrations of 15 (top) and 30 (bottom) mmol/kg. The two precursors were mixed at 80 °C and the samples were taken after 15 min at (1) 80 °C, (2) 100 °C, (3) 120 °C, (4) 140 °C, (5) 160 °C, (6) 180 °C, (7) 200 °C, (8) 220 °C, and (9) 240 °C.

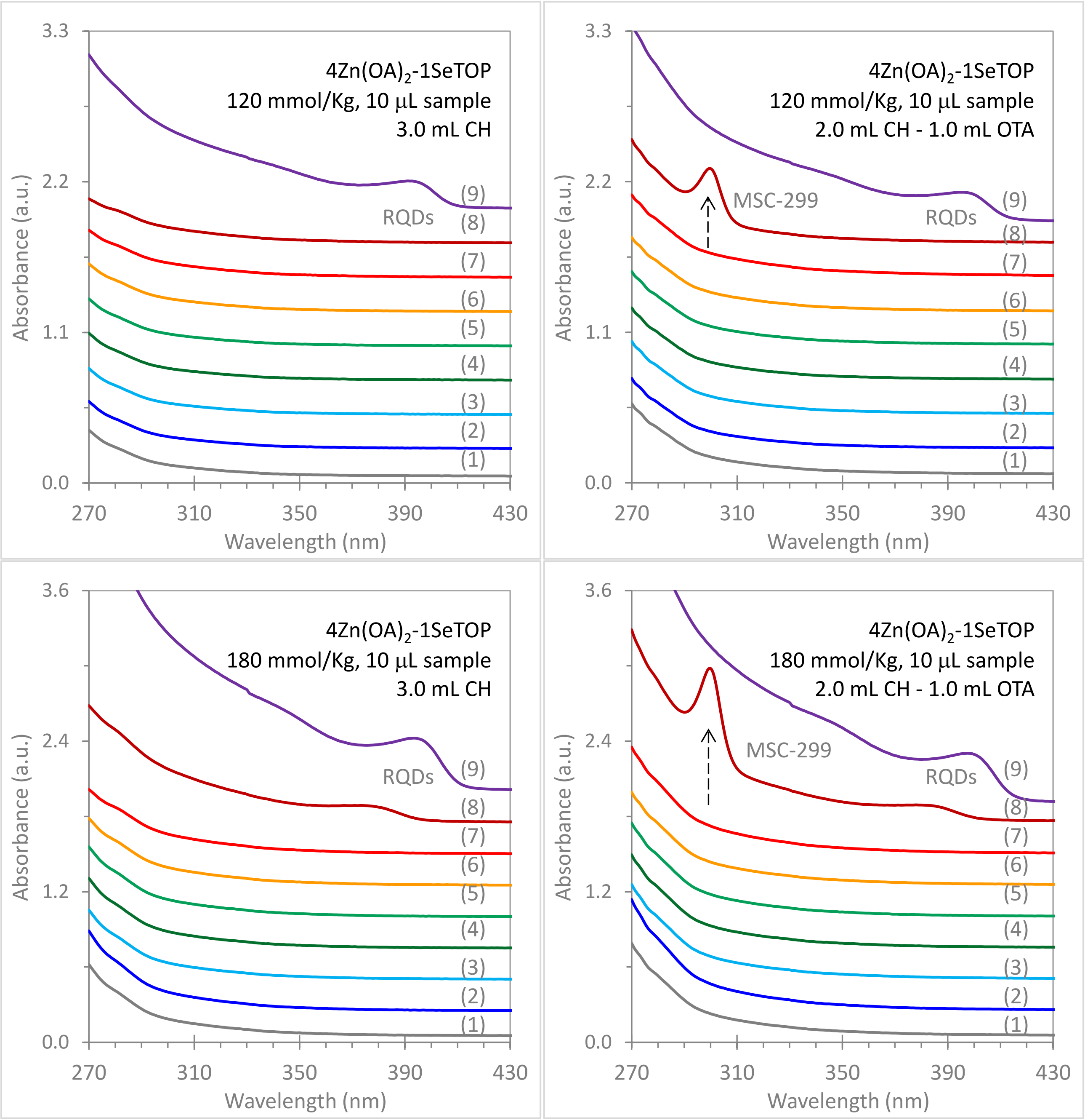

Figure S1-4. The evolution with temperature of the absorption of the as-prepared reaction product, similar to Figure S1-3a except the Se concentration and sample amount are different, as indicated.

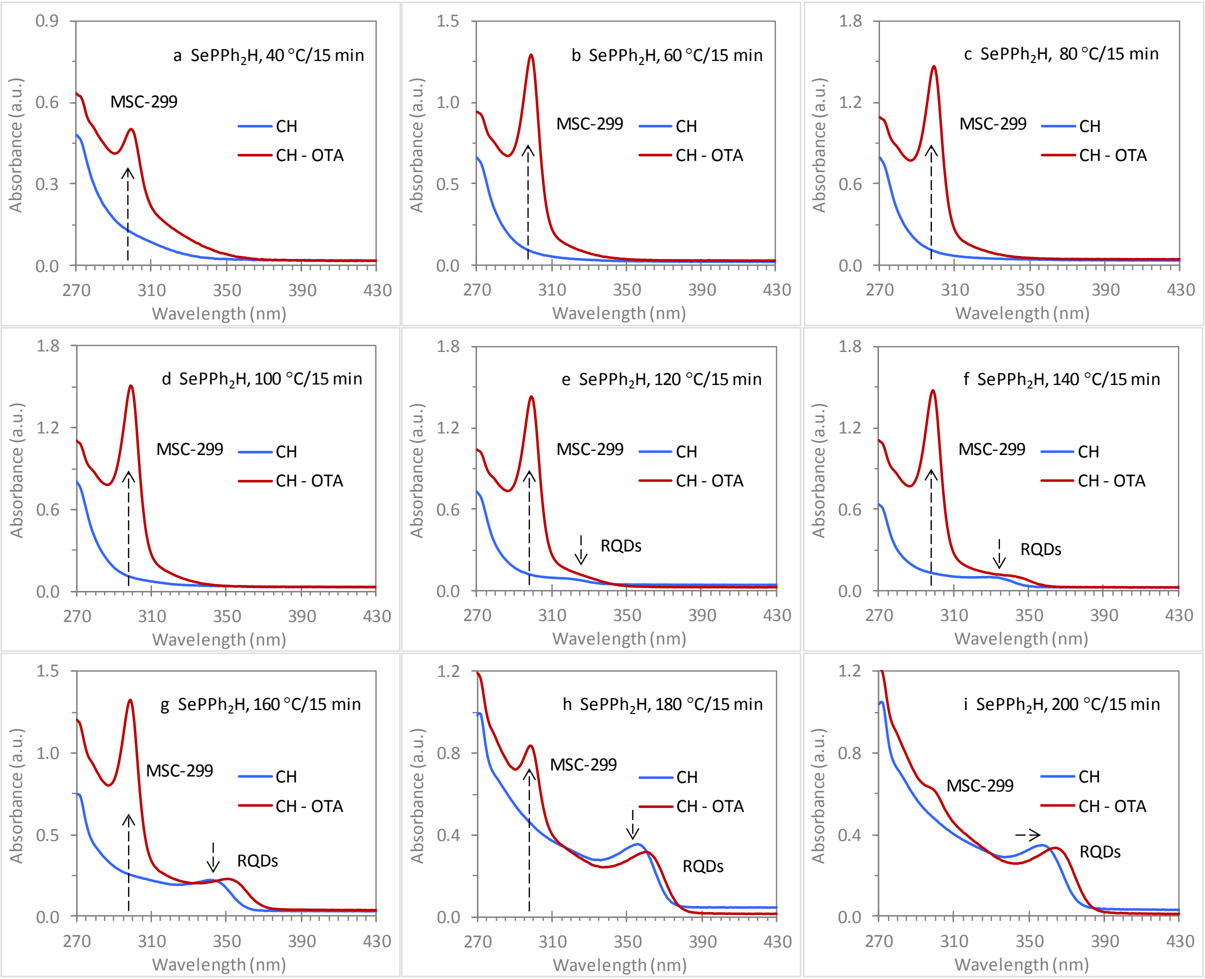

Figure S2. Comparison of the absorption spectra for each (15 μL) of the nine samples of the Reaction 3 batch collected after 15 min at (a) 40 °C (b) 60 °C, (c) 80 °C, (d) 100 °C, (e) 120 °C, (f) 140 °C, (g) 160 °C, (h) 180 °C, and (i) 200 °C. All the samples were then dispersed in 3.0 mL of CH (blue traces) and in the 2.0 mL CH – 1.0 mL OTA mixture (red traces).

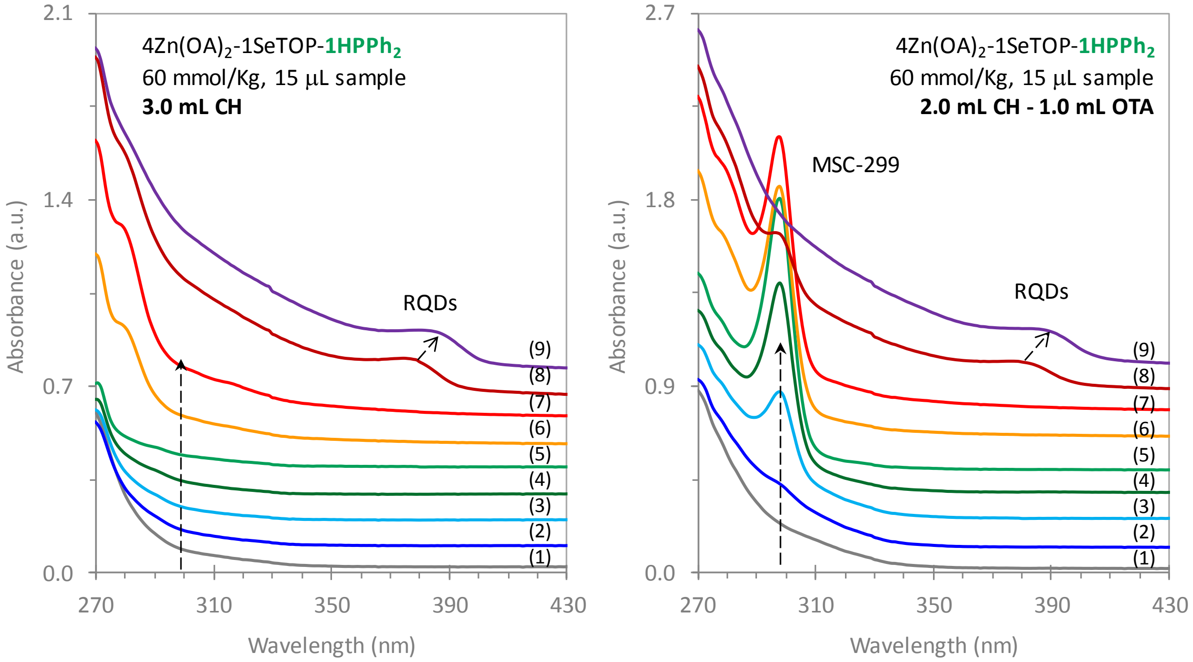

Figure S3-1. The evolution with temperature of the absorption of the samples (15 μL) dispersed in 3.0 mL of CH (left) and in the mixture of 2.0 mL CH – 1.0 mL OTA (right) from the reaction of $Zn(OA)_2$ + SeTOP + $HPPh_2$ associated with Figure 3. Here, the samples were taken after 15 min at (1) 80 °C, (2) 100 °C, (3) 120 °C, (4) 140 °C, (5) 160 °C, (6) 180 °C, (7) 200 °C, (8) 220 °C, and (9) 240 °C. The induction period prior to nucleation/growth of RQDs was observed to be below 180 °C (yellow spectrum (6)), and MSC-299 was detected in the 100 °C to 220 °C samples (right). For Reaction (4) with the Se feed concentration of 30 mmol/kg, a small population of MSC-299 was detected at 160 °C, as shown in Figure S3-4 (for 80 to 240 °C), S4-2a (at 140 °C), and S4-2b (at 180 °C). As we mentioned in the main text, the actual CMC value is related to the precursor.

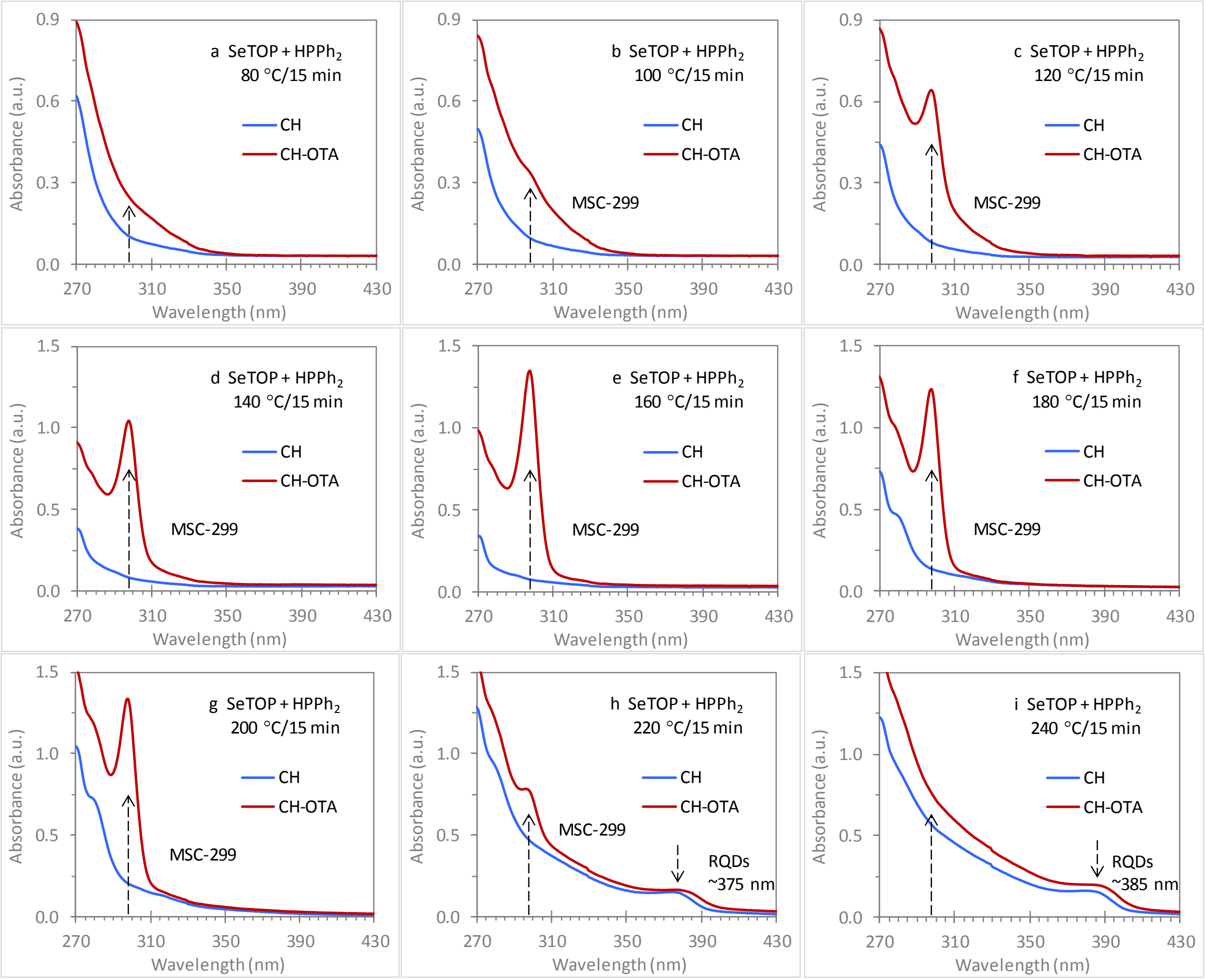

Figure S3-2. Comparison of the absorption spectra collected from each (15 μL) of the nine samples of the Figure 3 batch, when dispersed in CH (3.0 mL, blue traces) and in the CH - OTA mixture (2.0 mL CH + 1.0 mL OTA, red traces). The growth temperature of the nine samples was (a) 80 °C (b) 100 °C, (c) 120 °C, (d) 140 °C, (e) 160 °C, (f) 180 °C, (g) 200 °C, (h) 220 °C, and (i) 240 °C.

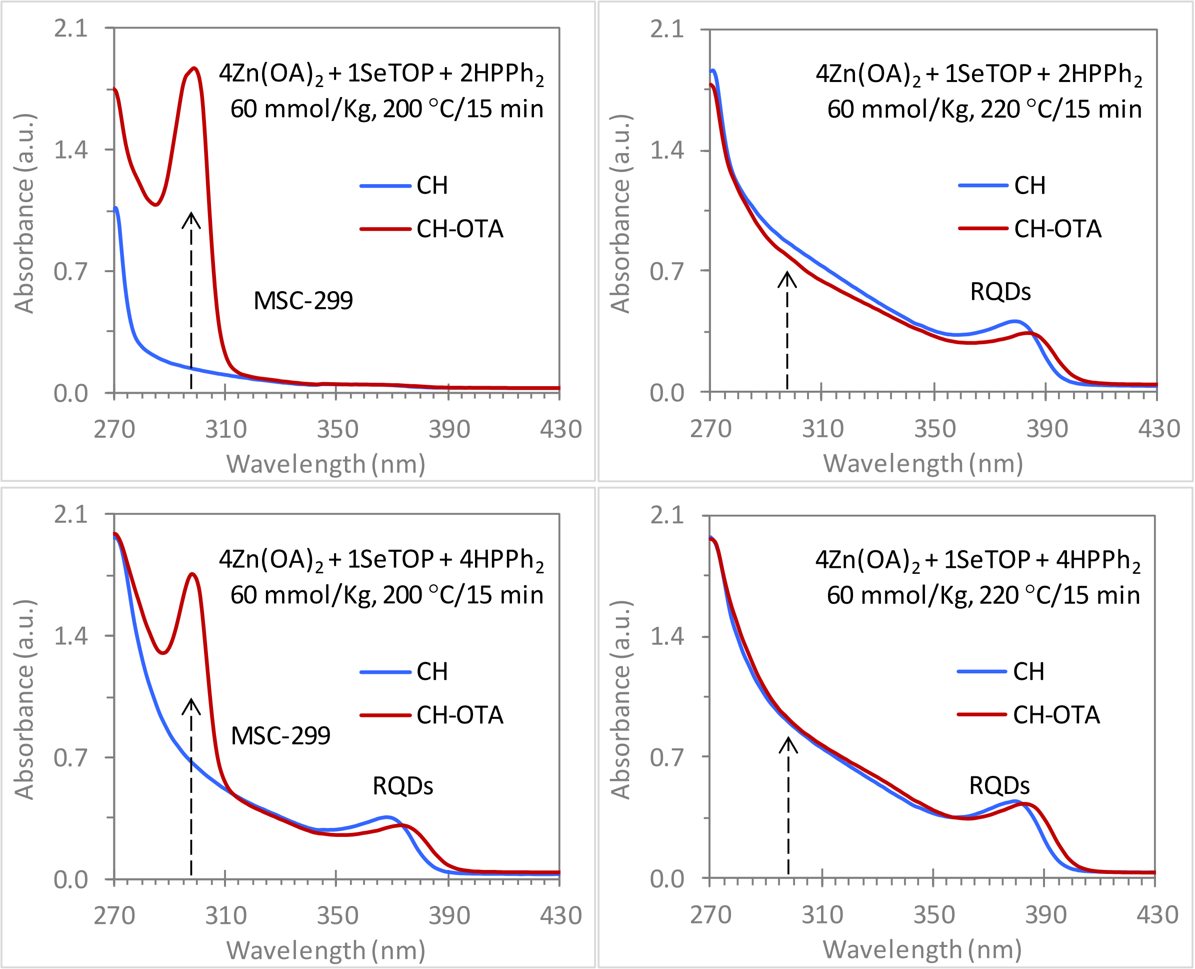

Figure S3-3. Comparison of the absorption spectra collected from samples (15 μL) taken from the reaction, $4Zn(OA)_2$ + 1SeTOP + $x$$HPPh_2$, with x = 2 (top panel) and x = 4 (bottom panel), dispersed in CH (3.0 mL, blue traces) and in the CH - OTA mixture (2.0 mL CH + 1.0 mL OTA, red traces). The Zn and Se precursors were mixed at 80 °C with the feed molar ratio of 4 to 1 and a Se concentration in ODE of 60 mmol/kg. The presence of RQDs was detected at 220 °C and 200 °C for x = 2 and 4, respectively. With x = 1 (Figures 3 and S3-1 and S3-2) or 2, the temperature required for nucleation and growth of RQDs was higher than 200 °C. Thus, the formation of IP-299 occurred within a large experimental window, which can be controlled to be in the induction period.

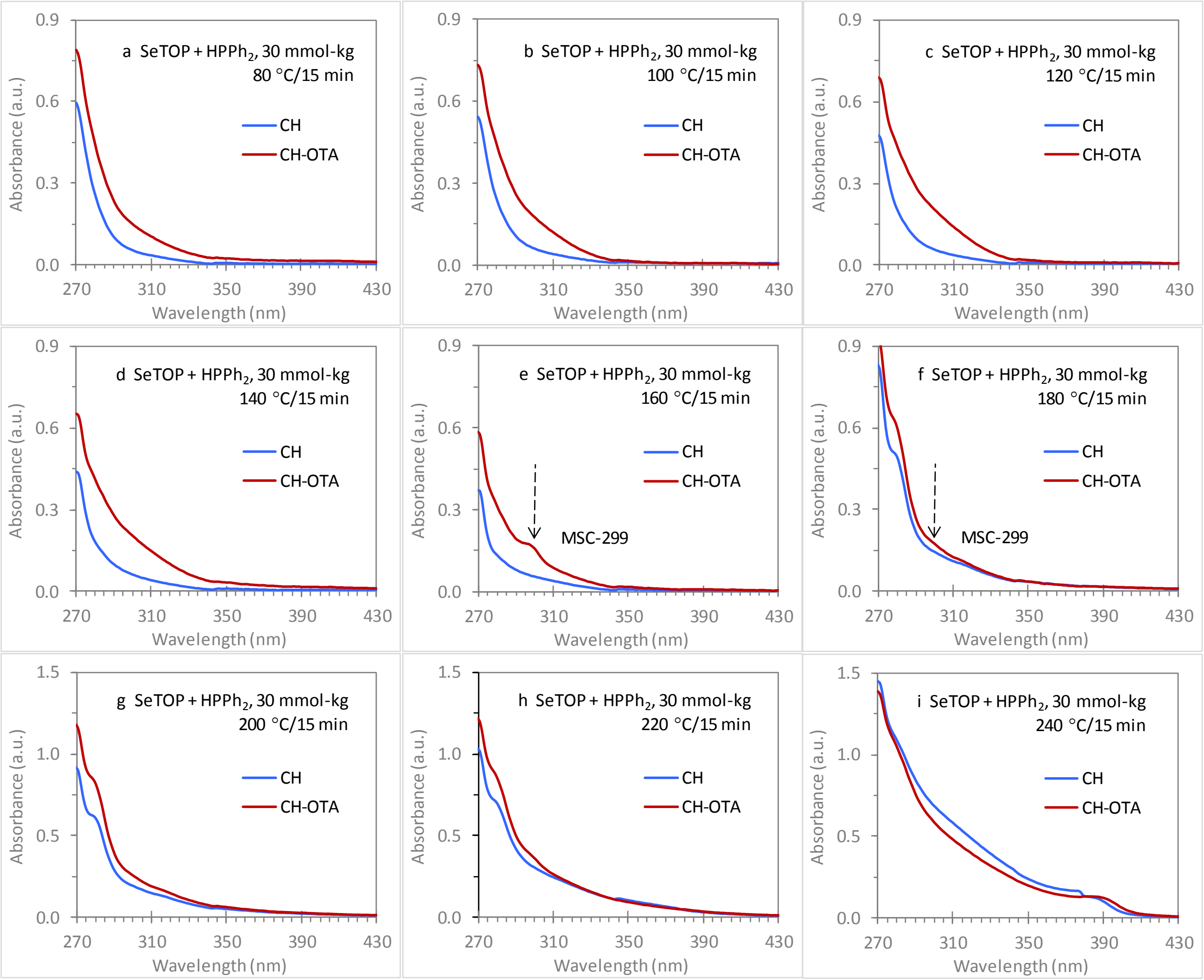

Figure S3-4. Comparison of the absorption spectra collected from the same product (30 μL) of the reaction, $Zn(OA)_2$ + SeTOP + $HPPh_2$, dispersed in CH (3.0 mL, blue traces) and in the CH - OTA mixture (2.0 mL CH + 1.0 mL OTA, red traces). A SeTOP solution and $HPPh_2$ were pre-mixed in a glove box with a feed molar ratio of 1 to 1. The Zn and Se precursors were mixed at 80 °C with the feed molar ratio of 4 to 1 and with a Se concentration in ODE of 30 mmol/Kg. Samples were taken when the reaction temperature of (a) 80 °C (b) 100 °C, (c) 120 °C, (d) 140 °C, (e) 160 °C, (f) 180 °C, (g) 200 °C, (h) 220 °C, and (i) 240 °C had been reached for 15 min as indicated. The growth temperature of the nine samples was from 80 °C to 240 °C as indicated.

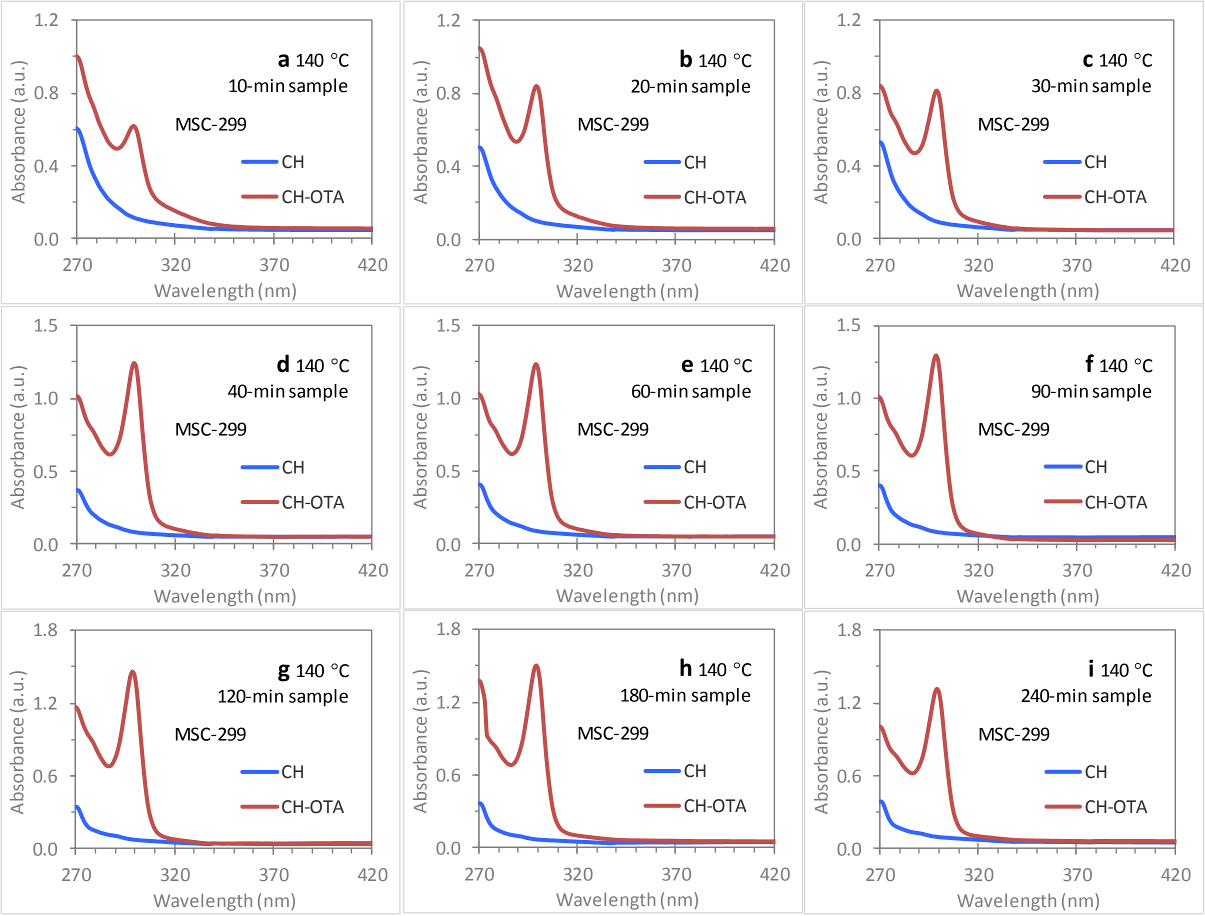

Figure S4-1a. Comparison of the absorption spectra collected from one sample (15 μL) in 3.0 mL of CH (blue traces) and in the 2.0 mL CH – 1.0 mL OTA mixture (red traces). The samples were from the reaction of $Zn(OA)_2$ + SeTOP + $HPPh_2$ (as for Figure 3), but at a constant growth temperature of 140 °C. The growth period at this temperature has been varied to be (a) 10 min (b) 20 min, (c) 30 min, (d) 40 min, (e) 60 min, (f) 90 min, (g) 120 min, (h) 180 min, and (i) 240 min, as indicated. The induction period extended to at least 240 min.

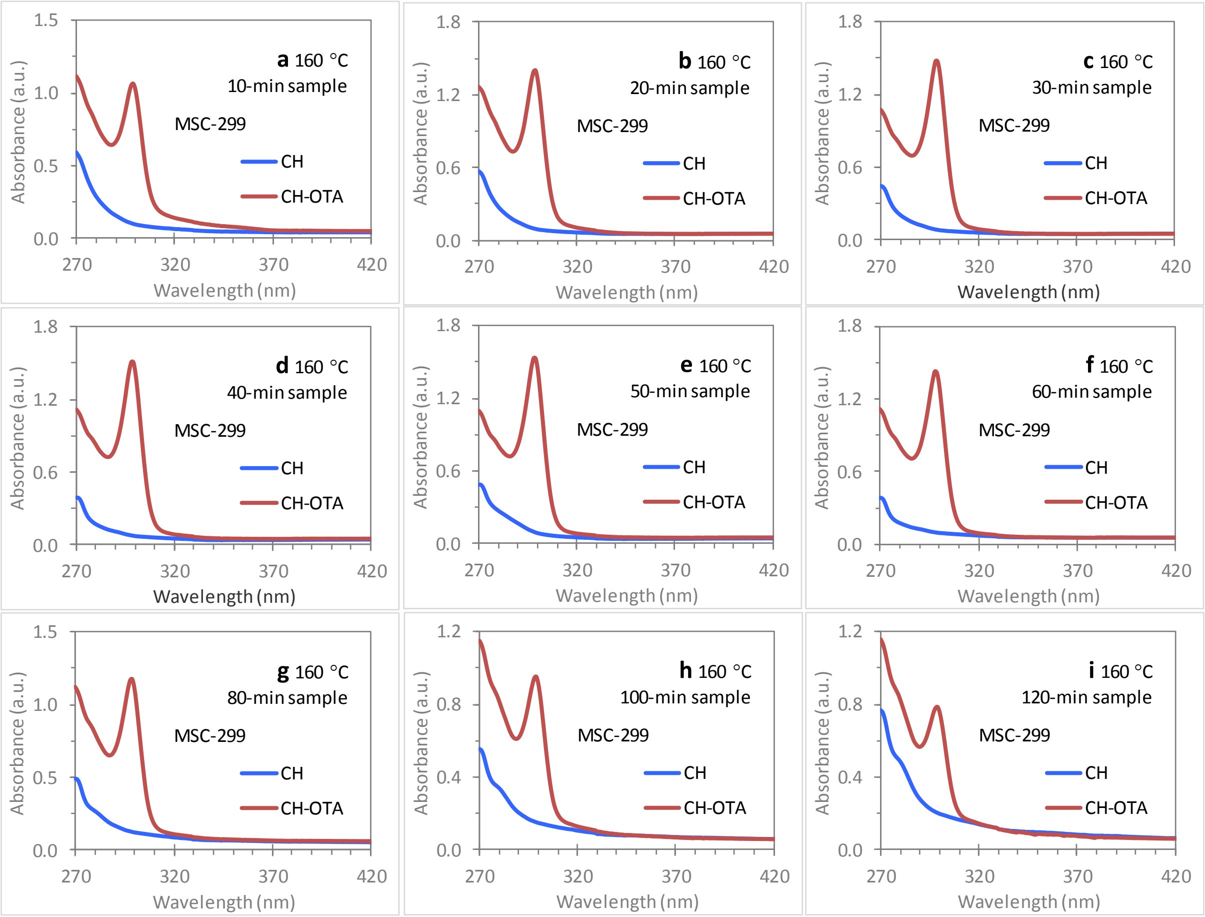

Figure S4-1b. Comparison of the absorption spectra collected from one sample (15 μL) in 3.0 mL of CH (blue traces) and in the 2.0 mL CH – 1.0 mL OTA mixture (red traces). The samples were from the reaction of $Zn(OA)_2$ + SeTOP + $HPPh_2$ (as for Figure 3), but at a constant growth temperature of 160 °C. The growth period at this temperature has been varied to be (a) 10 min (b) 20 min, (c) 30 min, (d) 40 min, (e) 50 min, (f) 60 min, (g) 80 min, (h) 100 min, and (i) 120 min, as indicated. The induction period extended to at least 100 min. For our innovative two-step approach to sole ensemble ZnSe MSC-299, we optimized the experimental conditions for each step. In particular, we performed the synthesis of IP-299 at relatively high temperatures and its structural transformation to MSC-299 at relatively low temperatures. For the first step, the use of a secondary phosphine (Reaction (4)) to have a practical window for the Zn-Se covalent bond formation in the induction period (via the proper control of the Se transfer from the tertiary phosphine to the secondary phosphine, SeTOP + $HPPh_2$ $\Leftrightarrow$ TOP + $SePPh_2H$) seemed to be the key. For the second step, the use of a primary amine instead of a secondary one (as shown by Figure S6-3) was critical.

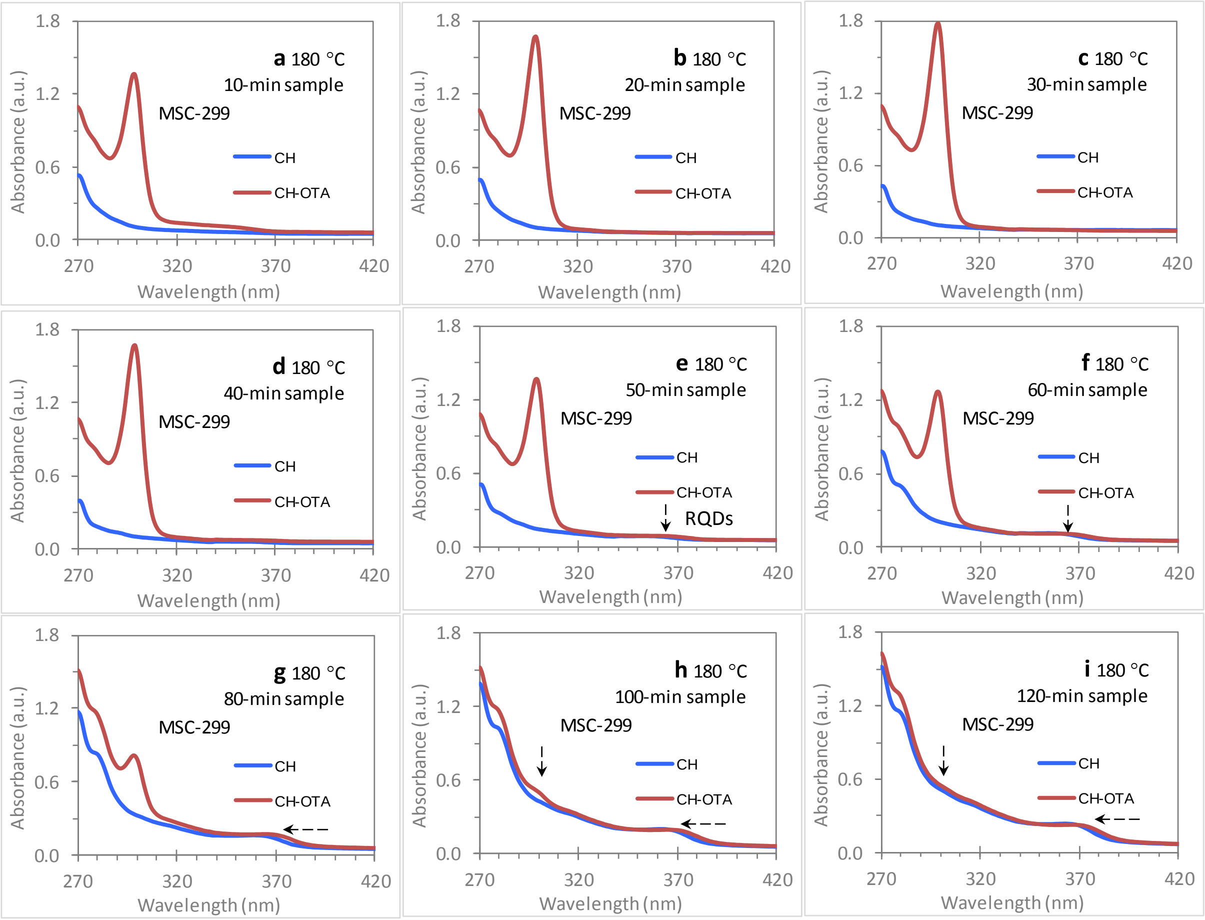

Figure S4-1c. Comparison of the absorption spectra collected from one sample (15 μL) in 3.0 mL of CH (blue traces) and in the 2.0 mL CH – 1.0 mL OTA mixture (red traces). The samples were from the reaction of $Zn(OA)_2$ + SeTOP + $HPPh_2$ (as for Figure 3), but at a constant growth temperature of 180 °C. The growth period at this temperature has been varied to be (a) 10 min (b) 20 min, (c) 30 min, (d) 40 min, (e) 50 min, (f) 60 min, (g) 80 min, (h) 100 min, and (i) 120 min, as indicated. The induction period extended to at least 40 min.

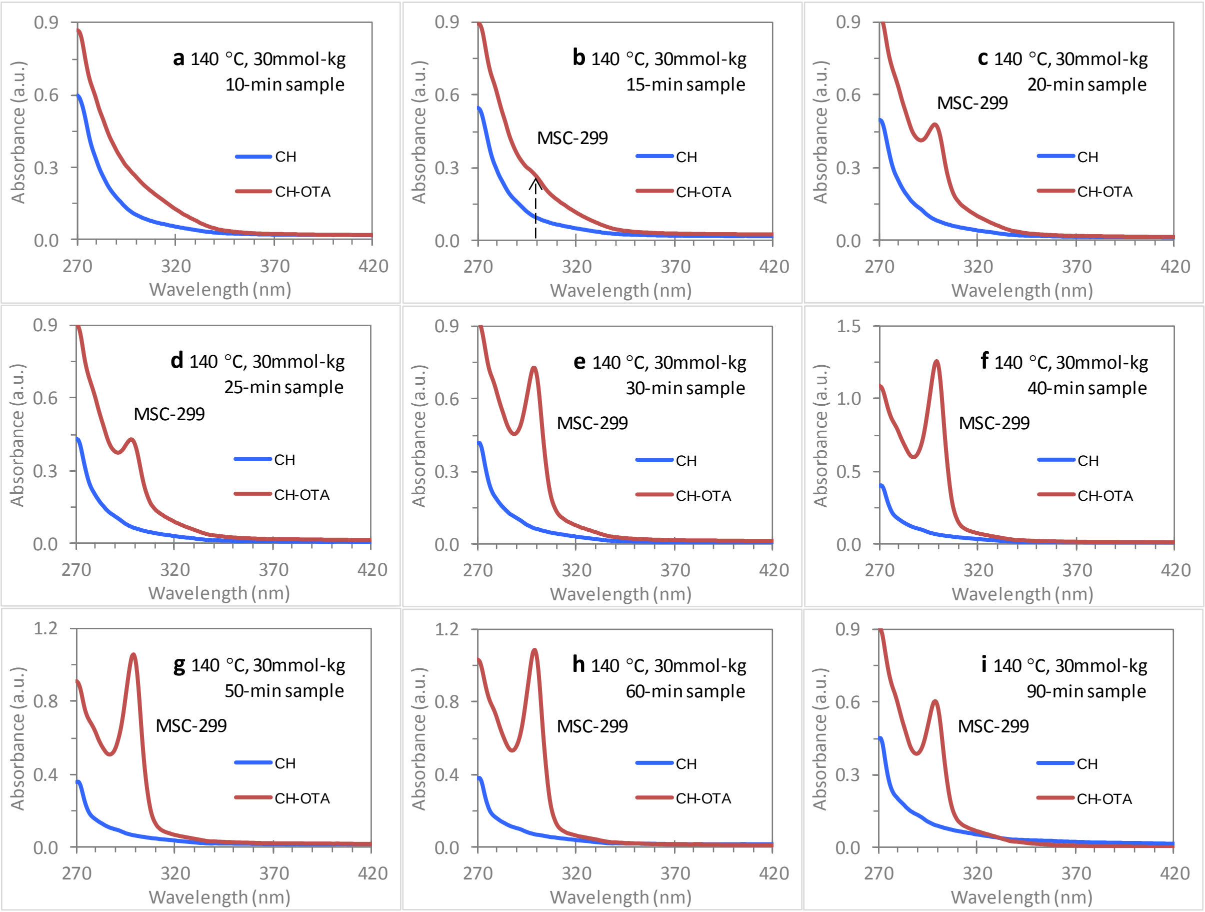

Figure S4-2a. Comparison of the absorption spectra collected from one sample (30 μL) in 3.0 mL of CH (blue traces) and in the mixture of 2.0 mL CH – 1.0 mL OTA (red traces). The samples were from a reaction (identical to that for Figure S3-4), $Zn(OA)_2$ + SeTOP + $HPPh_2$ but at a constant growth temperature at 140 °C. The growth periods are set to be (a) 10 min (b) 15 min, (c) 20 min, (d) 25 min, (e) 30 min, (f) 40 min, (g) 50 min, (h) 60 min, and (i) 90 min.

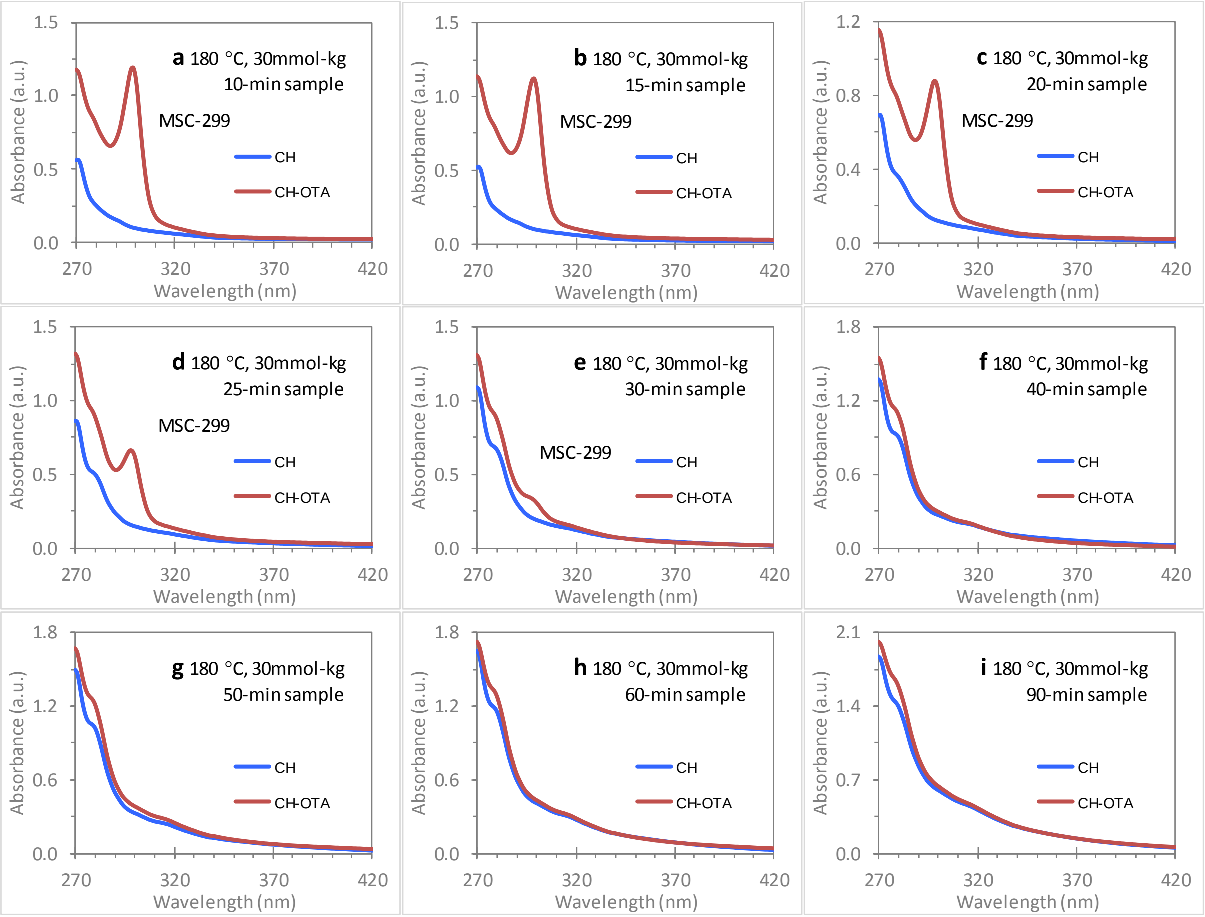

Figure S4-2b. Comparison of the absorption spectra collected from one sample (30 μL) in 3.0 mL of CH (blue traces) and in the mixture of 2.0 mL CH – 1.0 mL OTA (red traces). The samples were from a reaction (identical to that for Figure S3-4), $Zn(OA)_2$ + SeTOP + $HPPh_2$ but at a constant growth temperature at 180 °C. The growth periods are set to be (a) 10 min (b) 15 min, (c) 20 min, (d) 25 min, (e) 30 min, (f) 40 min, (g) 50 min, (h) 60 min, and (i) 90 min.

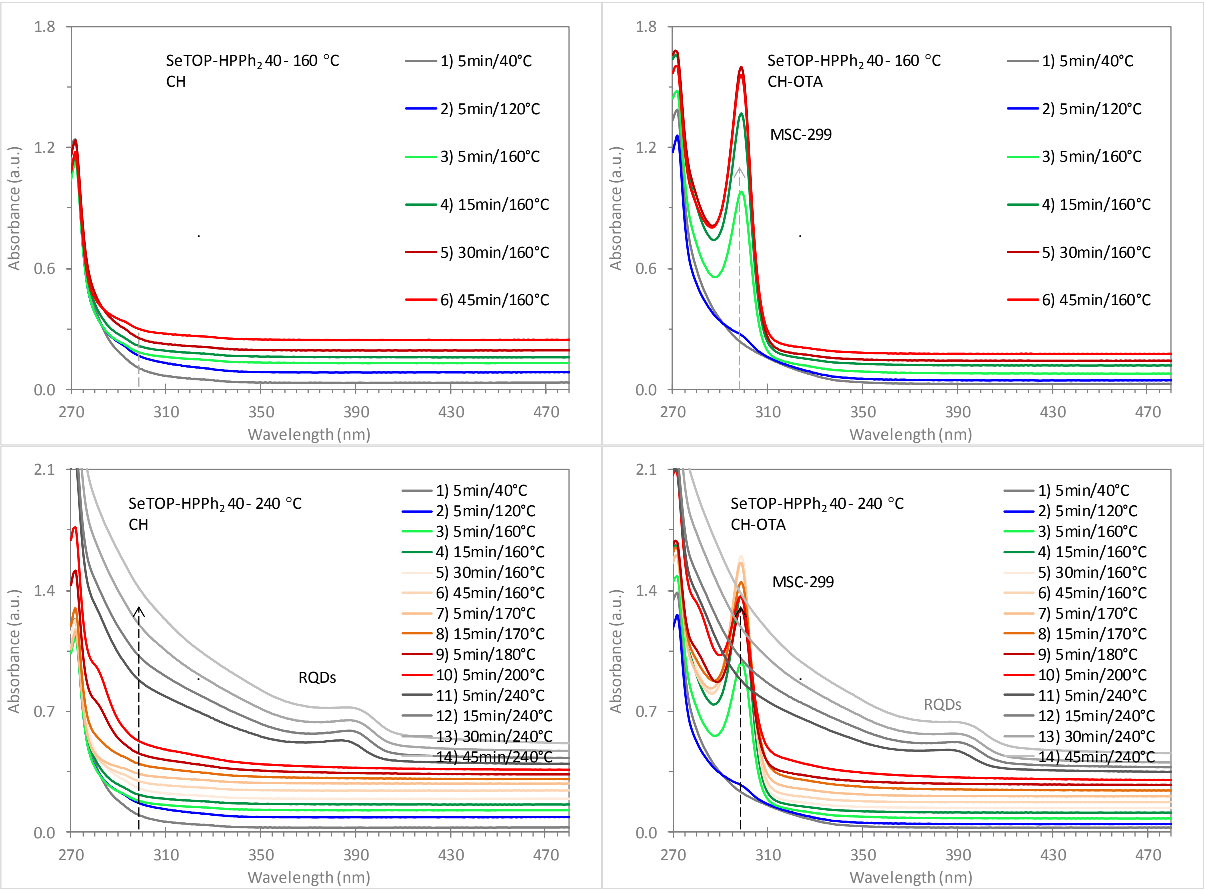

Figure S5-1a. The evolution with temperature of the absorption of the as-prepared reaction product from one reaction of $Zn(OA)_2$ + SeTOP + $HPPh_2$, which were prepared for the MS study with results shown in Figure 5. A SeTOP solution and $HPPh_2$ were pre-mixed in a glove box with a feed molar ratio of 1 to 4. The precursors were mixed at 40 °C with a Zn to Se feed molar ratio of 4 to 1 and a Se concentration in ODE of 30 mmol/Kg. Samples were taken when the reaction temperatures were reached and after a specific time periods (as indicated). The samples (30 µL) were dispersed in 3.0 mL of CH (left panel) and in the 2.0 mL CH – 1.0 mL OTA mixture (right panels). ZnSe MSC-299 was not apparently presented in the 40 °C sample, but was detected in the 160 °C samples in the CH - OTA mixture (right). The induction period was apparently below 200 °C (red spectra of Sample 10).

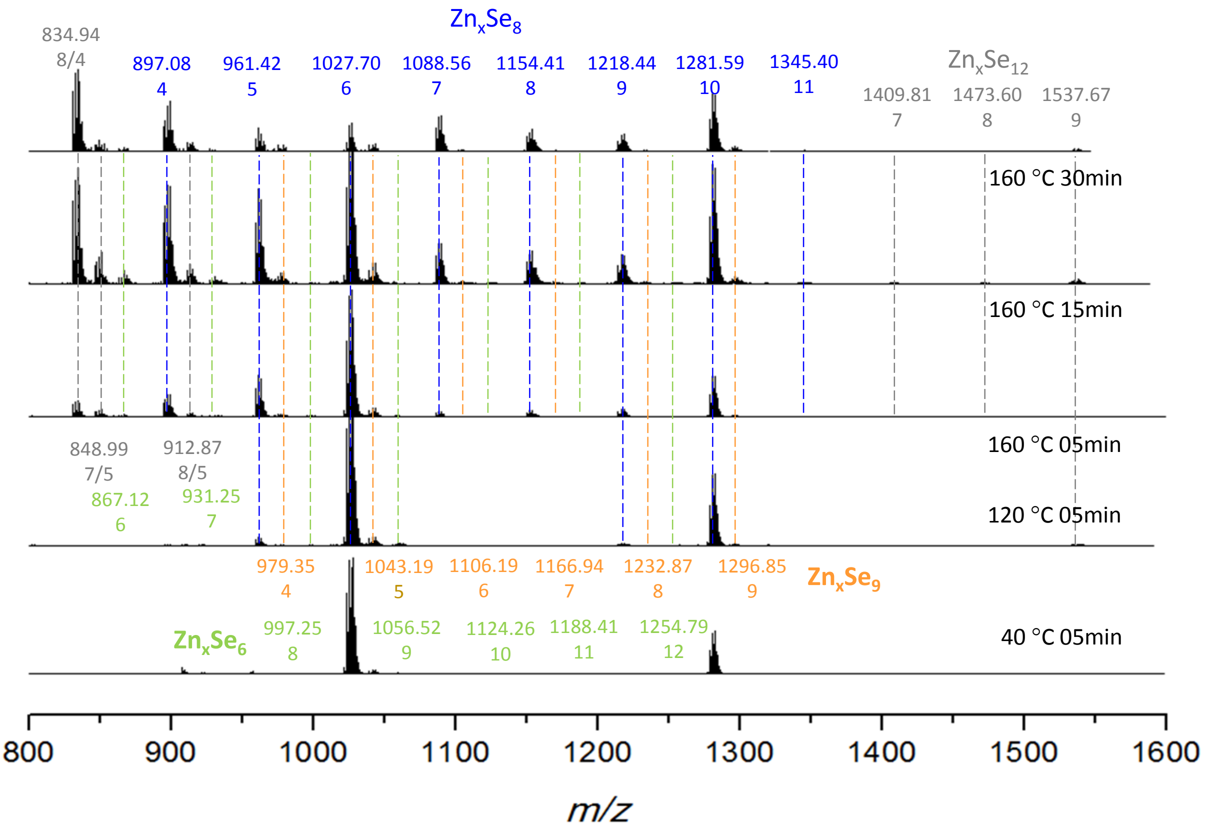

Figure S5-1b. ESI-MS study of the five samples from the reaction of $Zn(OA)_2$ + SeTOP + $HPPh_2$ associated with Figure S5-1a. The results were from a negative-ion mode. ZnSe MSC-299 was not detected in the 40 °C sample, but was observed for the other four samples dispersed in the CH - OTA mixture. The detection of the cluster fragments, including Zn_xSe_6 (green color), Zn_xSe_8 (blue color) and Zn_xSe_9 (yellow color), indicates Zn-Se bonds have been formed in these samples.

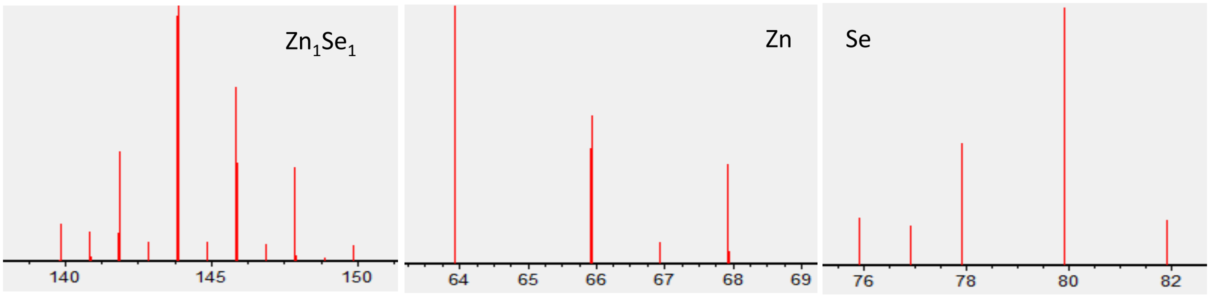

Figure S5-1c. Calculated isotope distribution of ZnSe (left), together with the isotope distribution of Zn (middle) and Se (right), from the Isopro software with Lorentz fitting.

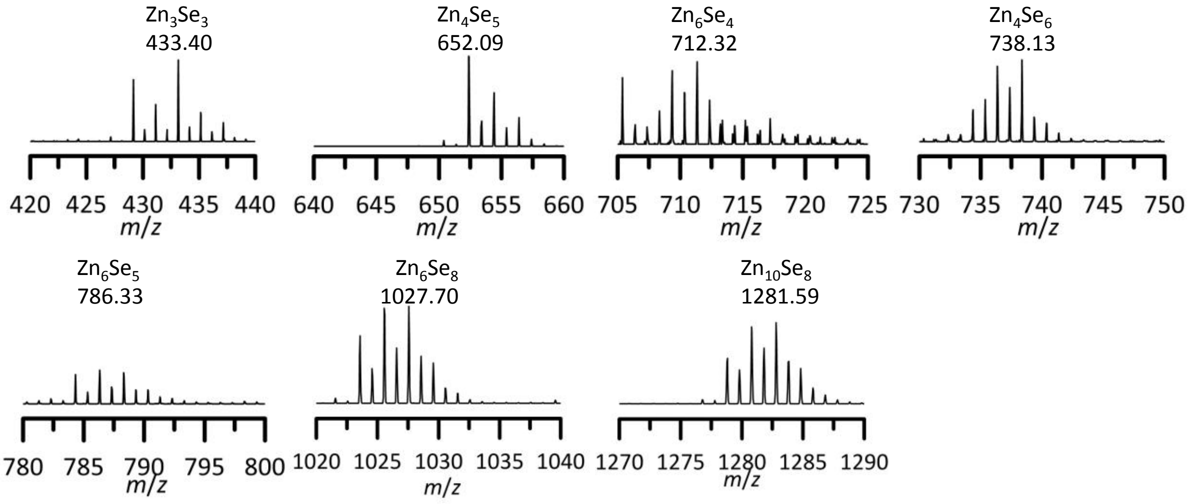

Figure S5-1d. Summary of the cluster fragments detected in the m/z 300–1600 region for the 40 °C/5 min sample.

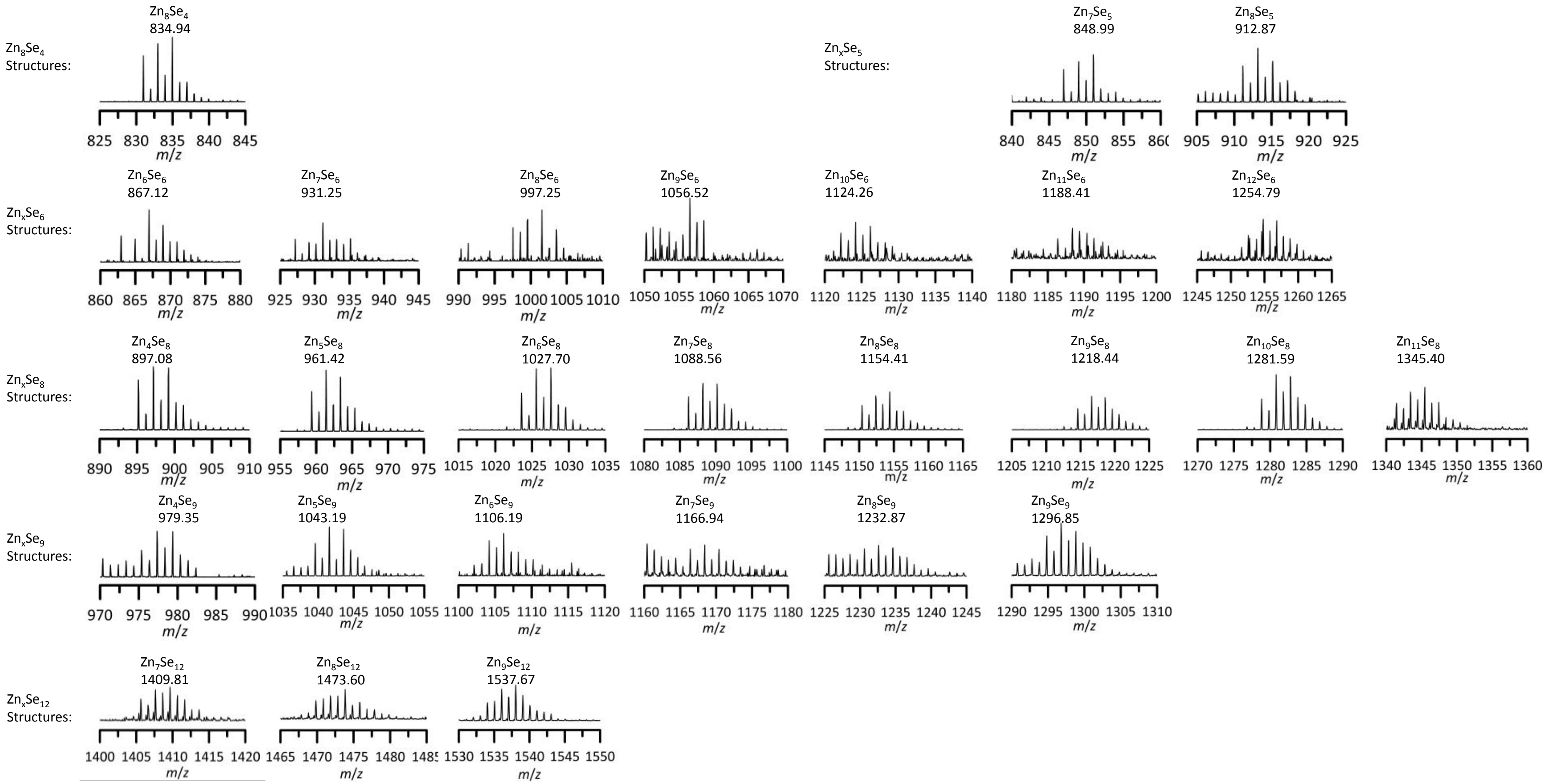

Figure S5-1e. Summary of the cluster fragments detected in the m/z 800–1600 region for the 160 °C/15 min sample.

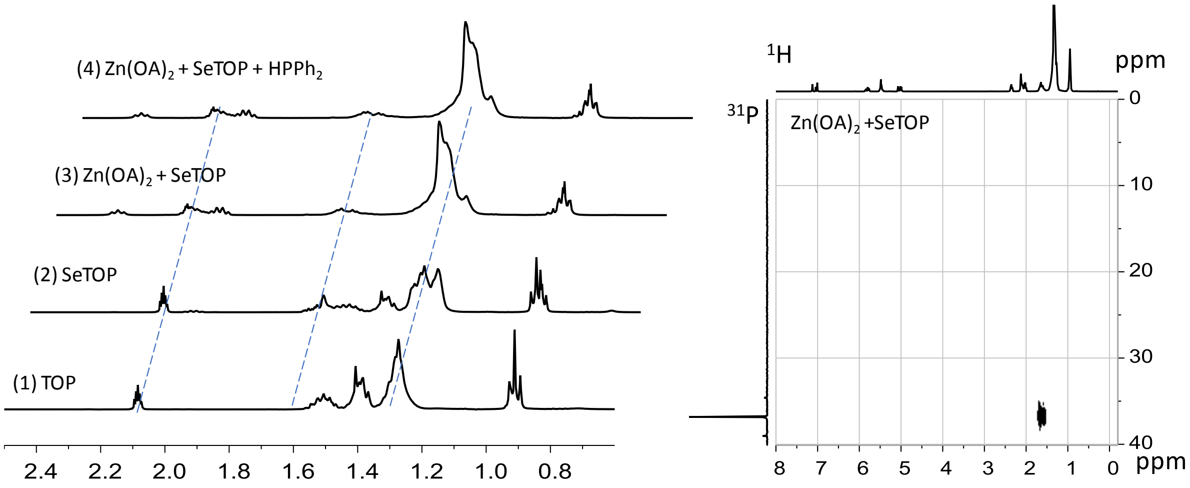

Figure S5-2. (left) ^{1}H NMR spectra of TOP (1), SeTOP (2), $Zn(OA)_2$ + SeTOP (3) and $Zn(OA)_2$ + SeTOP + $HPPh_2$ (4) with 0.03% tetramethylsilane (TMS) in d_8- toluene (99.5%, Acros) as an internal standard. (right) ^{1}H - ^{31}P heteronuclear multiple bond correlation spectroscopy (HMBC) of the $Zn(OA)_2$ + SeTOP sample in d_8-toluene (99.5%, CIL). The ^{1}H NMR spectra were taken using an Agilent 400-MR DD2, and the ^{1}H NMR HMBC spectrum were collected using a Bruker Avance III (400MHz). For the four mixtures studied in 0.50 mL of d_8-Toluene, they were prepared with (1) 3.0 mg of TOP, (2) 5.0 mg of SeTOP stock solution, (3) 5.0 mg of SeTOP stock solution and 33.5 mg of $Zn(OA)_2$ stock solution (with a molar ratio of 1 : 4), and (4) 5.0 mg SeTOP stock solution, 33.5 mg $Zn(OA)_2$ stock solution and 1.1 mg $HPPh_2$ (with a molar ratio of 1 : 4 : 4).

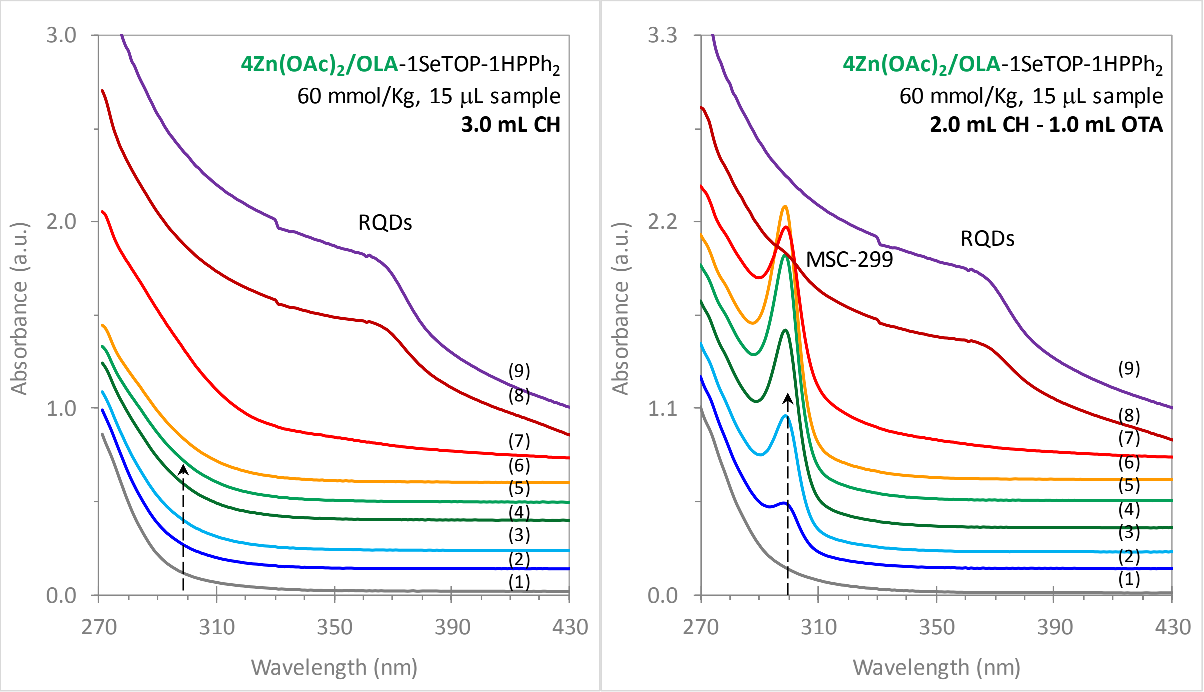

Figure S6-1a. The evolution with temperature of the absorption of the samples (15 μL) dispersed in cyclohexane (CH, 3.0 mL left panel) and in the CH (2.0 mL) + OTA (1.0 mL) mixture (right panel), for the reaction indicated. To prepare the Zn precursor $Zn(OAc)_2$/OLA, similar to the preparation of $Cd(OAc)_2$/OLA,[1] a mixture of the $Zn(OAc)_2$ (0.2202 g, 1.20 mmol) and OLA (5.3 mL, oleylamine 70%, Aldrich) was placed in a 50 mL three-necked flask. Following the same procedure as used for the $Zn(OA)_2$ preparation, the mixture was heated to 80 °C under a N_2 atmosphere, then evacuated and backfilled with N_2 gas. This procedure was repeated three times. Next, under a N_2 atmosphere, the mixture was heated up to 120 °C, then held under vacuum for 2 hrs, followed by cooling under a N_2 atmosphere to 80 °C. A mixture of SeTOP (330 μL, 0.30 mmol) and $HPPh_2$ (52 μL, 0.30 mmol) and OLA (0.5 mL) was added to achieve a total weight for the reaction mixture of 5.000 g. The reaction flask was evacuated and then backfilled with N_2 gas, which was repeated three times. Under a N_2 atmosphere, the reaction mixture was heated from 80 °C to 200 °C with a 20 °C interval; the samples were removed after 15 min at (1) 80 °C, (2) 100 °C, (3) 120 °C, (4) 140 °C, (5) 160 °C, (6) 180 °C, (7) 200 °C, (8) 220 °C, and (9) 240 °C.

[1] M. Liu, K. Wang, L. Wang, S. Han, H. Fan, N. Rowell, J. A. Ripmeester, R. Renoud, F. Bian, J. Zeng, K. Yu, *Nat. Commun.* **2017**, *8*, 15467.

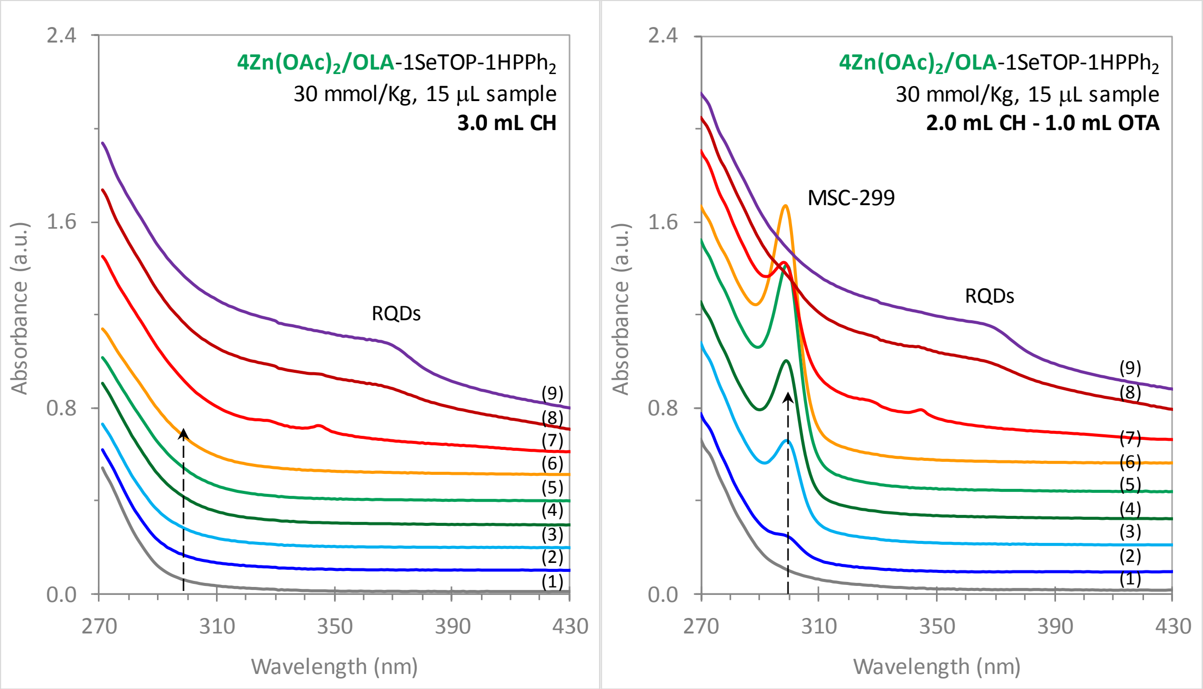

Figure S6-1b. The evolution with temperature of the absorption of the as-prepared reaction product (30 μL) dispersed in 3.0 mL of CH (left) and in the 2.0 mL CH – 1.0 mL OTA mixture (right) from the reaction, $4Zn(OAc)_2/OLA + 1SeTOP + 1HPPh_2$. This reaction consisted of $Zn(OAc)_2$ (0.1101 g, 0.60 mmol) and OLA (5.3 mL), together with SeTOP (165 μL, 0.15 mmol) and $HPPh_2$ (26 μL, 0.15 mmol) and OLA (0.5 mL), making the total weight of the reaction mixture to be 5.000 g. Samples were removed after 15 min at (1) 80 °C, (2) 100 °C, (3) 120 °C, (4) 140 °C, (5) 160 °C, (6) 180 °C, (7) 200 °C, (8) 220 °C, and (9) 240 °C. MSC-299 was detected in the 100 °C to 200 °C samples (right). The nucleation/growth of RQDs was observed to occur at temperature higher than 200 °C.

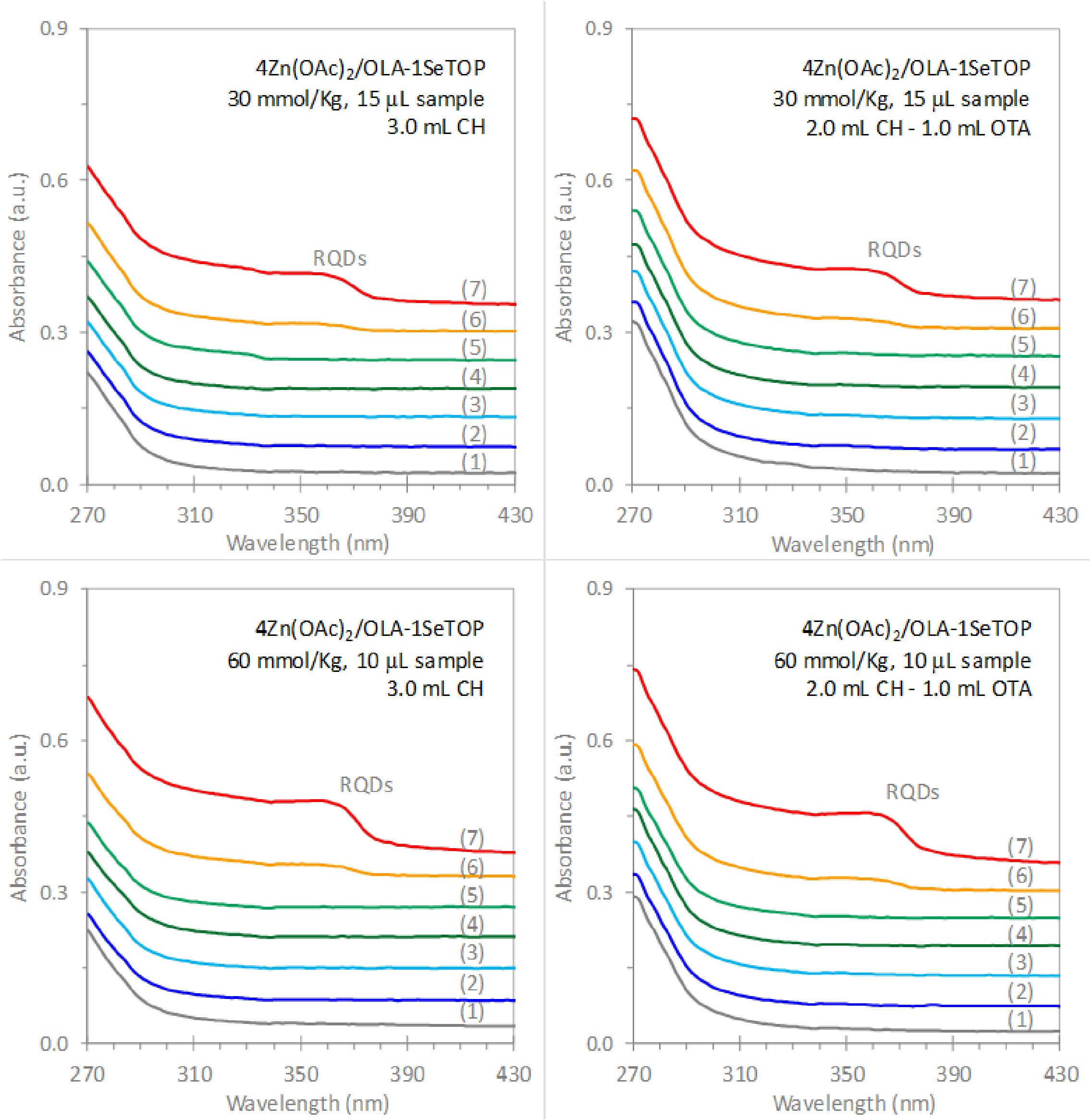

Figure S6-2a. The evolution with temperature of the absorption of the as-prepared reaction products from the reaction of $Zn(OAc)_2$/OLA + SeTOP with the feed molar ratio of 4Zn to 1Se and the Se concentration of 30 (top) and 60 (bottom) mmol/Kg. 15 μL (top) and 10 μL (bottom) samples were dispersed in cyclohexane (CH, 3.0 mL left) and in the mixture of CH (2.0 mL) and OTA (1.0 mL) (right). The two reactions consisted of $Zn(OAc)_2$ (0.1101 g, 0.60 mmol) and OLA (5.3 mL/ 5.1 mL for the top/bottom panels), together with SeTOP (165 μL, 0.15 mmol for the top and 330 μL, 0.30 mmol for the bottom panels) and OLA (0.5 mL), with a total weight of the reaction mixture of 5.000 g. Samples were extracted after 15 min at (1) 80 °C, (2) 100 °C, (3) 120 °C, (4) 140 °C, (5) 160 °C, (6) 180 °C and (7) 200 °C.

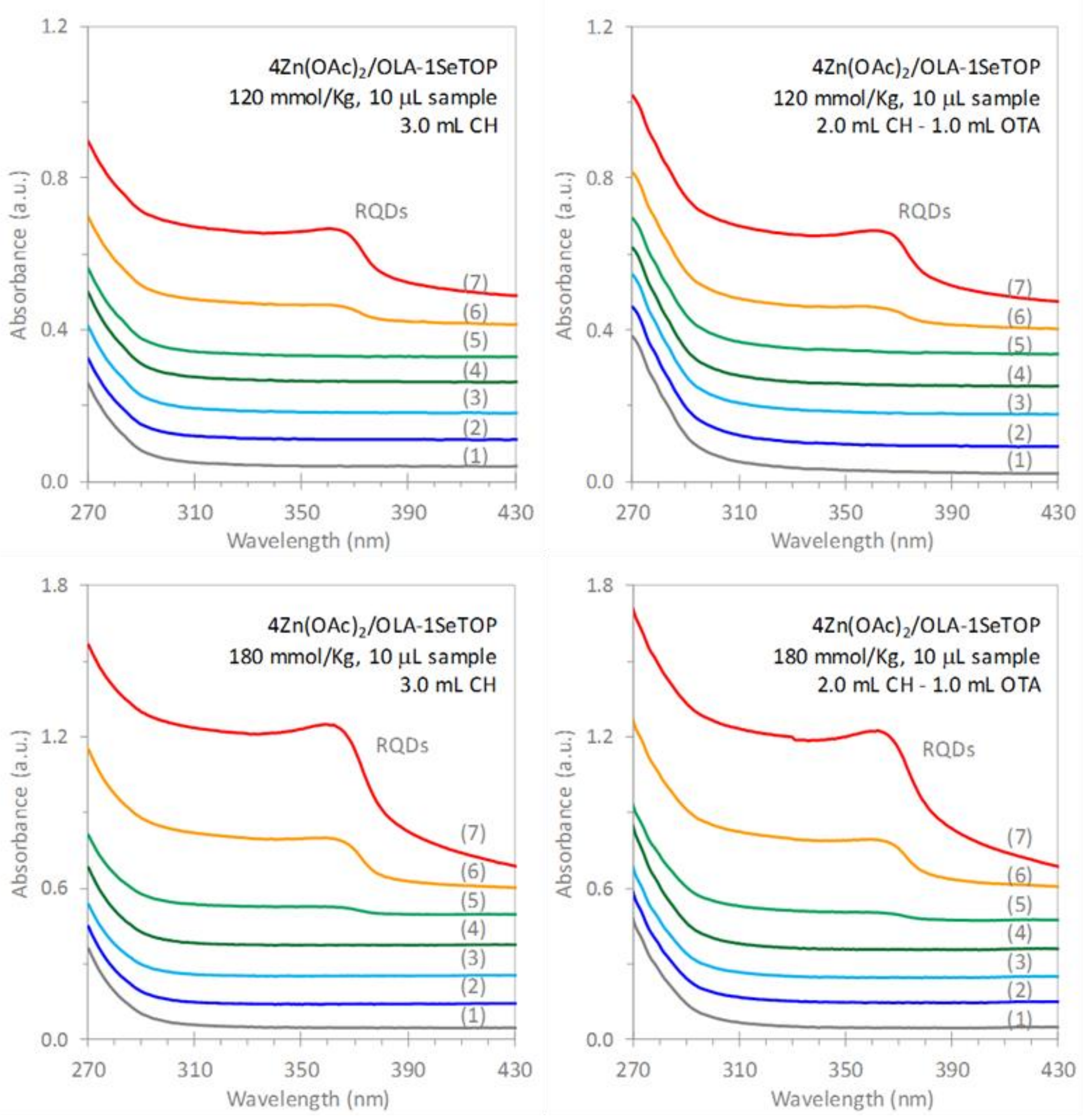

Figure S6-2b. The evolution with temperature of the absorption of the as-prepared reaction product (10 µL) dispersed in cyclohexane (CH, 3.0 mL left) and in the mixture of CH (2.0 mL) and OTA (1.0 mL) (right), from the reactions similar to those of Figure S6-2a with the Se concentrations as indicated.

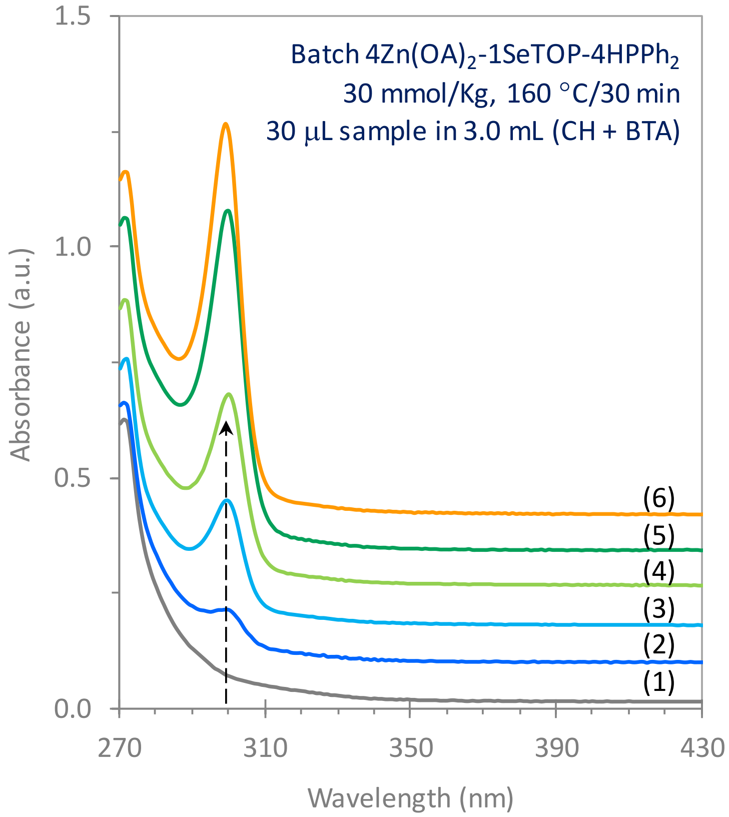

Figure S6-3a. The presence of MSC-299 in the CH-BTA (butylamine (99.5%, Aldrich)) dispersions. Different amounts of BTA (200 μL (1), 400 μL (2), 450 μL (3), 500 μL (4), 800 μL (5) and 1000 μL (6)) were used in the 3 mL of the CH-BTA mixtures. The 160 °C/30 min sample (30 μL) was from the $4Zn(OA)_2$ + 1SeTOP + $4HPPh_2$ reaction. We expect that the primary amines BTA and OTA play a similar role for the structural transformation from IP-299 to MSC-299. It was pointed out that the proton bonded to N of a primary amine probably played a critical role in the CdTe IP-371 ⇒ MSC-371 transformation;[1] the present results is in agreement with the hypothesis. We are actively exploring the role of primary amines on the molecular level for the formation of MSCs from IPs.

[1] M. Liu, K. Wang, L. Wang, S. Han, H. Fan, N. Rowell, J. A. Ripmeester, R. Renoud, F. Bian, J. Zeng, K. Yu, *Nat. Commun.* **2017**, *8*, 15467.

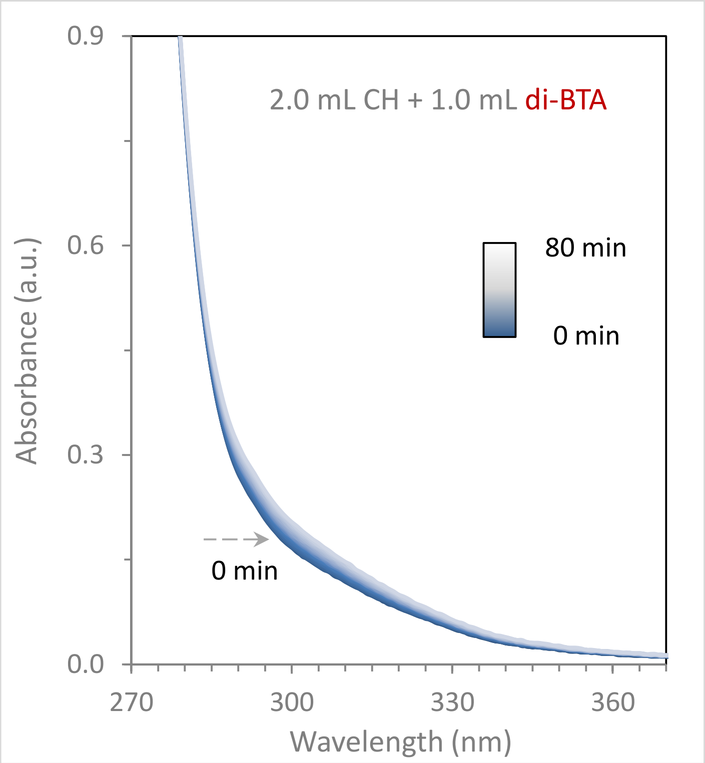

Figure S6-3b. The absorption spectroscopy study of the 160 °C/30 min sample (30 μL) from the $4Zn(OA)_2$ + 1SeTOP + $4HPPh_2$ reaction dispersed in a mixture of 2.0 mL of CH and 1.0 mL of di-*n*-butylamine (di-BTA, 99.5%, Aldrich). With the secondary amine in the dispersion, there is no MSC-299 detected. We are exploring the stereo-chemical control of the IP to MSC structural transformation.

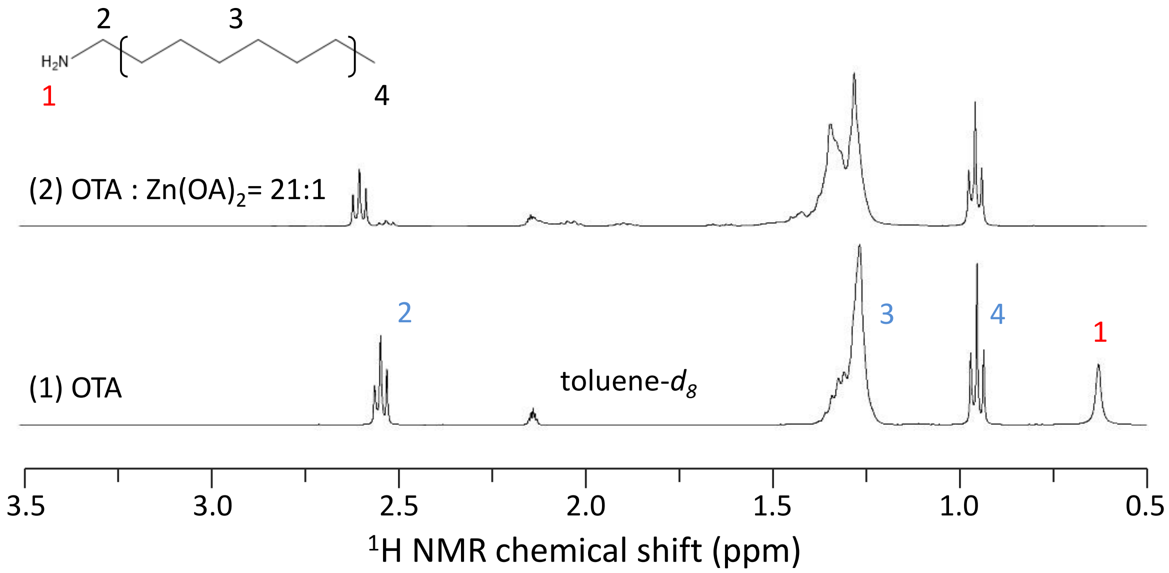

Figure S6-4a. Preliminary ^{1}H NMR spectra collected to help us understand the role of a primary amine added in the structural transformation from IP-299 to MSC-299. The NMR spectra illustrate that the chemical shift of the proton (labeled as number 1) bonded to the nitrogen atom of the OTA molecule exhibits down-field shift in the mixture (trace 2), as compared to that of a free OTA (trace 1). The shift is similar to what detected for those Cd-amine mixtures.[1] Such down field shift should be due to the interaction between a primary amine molecule and a Zn precursor molecule. Here, we used OTA (commercial) (1) and the mixtures of OTA and our Zn precursor $Zn(Ac)_2$·with the molar ratios of 21 : 1 (2). The mixtures were prepared by the addition of OTA to toluene-d_8 and then the Zn precursor was added. The OTA concentrations in toluene were about 20 mg OTA (1) and 2 mg OTA (2) in 1 mL toluene (1). We believe that it is this H (labeled as 1 bond to N) that interacts with Zn and plays a role in the IP-299 to MSC-299 structural transformation. The assignment follows close after that for Supporting Information Figure 12.[1] The peak at ~ 2.1 ppm can be attributed to d_8-toluene.[2]

[1] M. Liu, K. Wang, L. Wang, S. Han, H. Fan, N. Rowell, J. A. Ripmeester, R. Renoud, F. Bian, J. Zeng, K. Yu, *Nat. Commun.* **2017**, *8*, 15467.

[2] G. R. Fulmer, A. J. M. Miller, N. H. Sherden, H. E. Gottlieb, A. Nudelman, B. M. Stoltz, J. E. Bercaw, K. I. Goldberg, *Organometallics* **2010**, *29*, 2176.

.

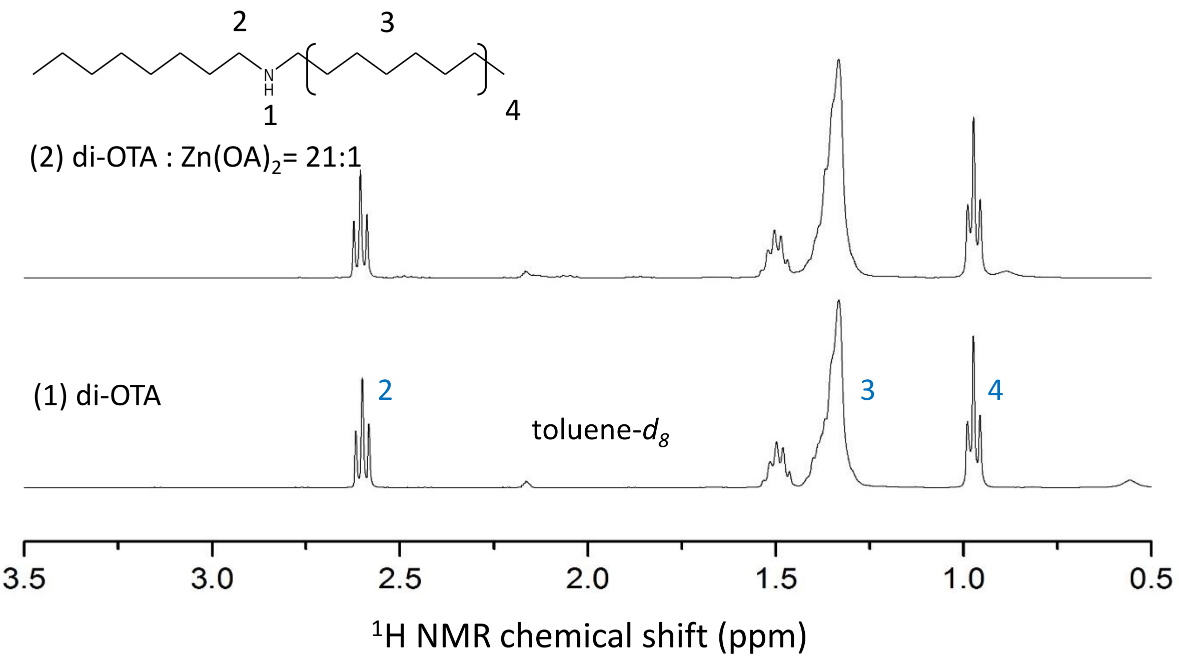

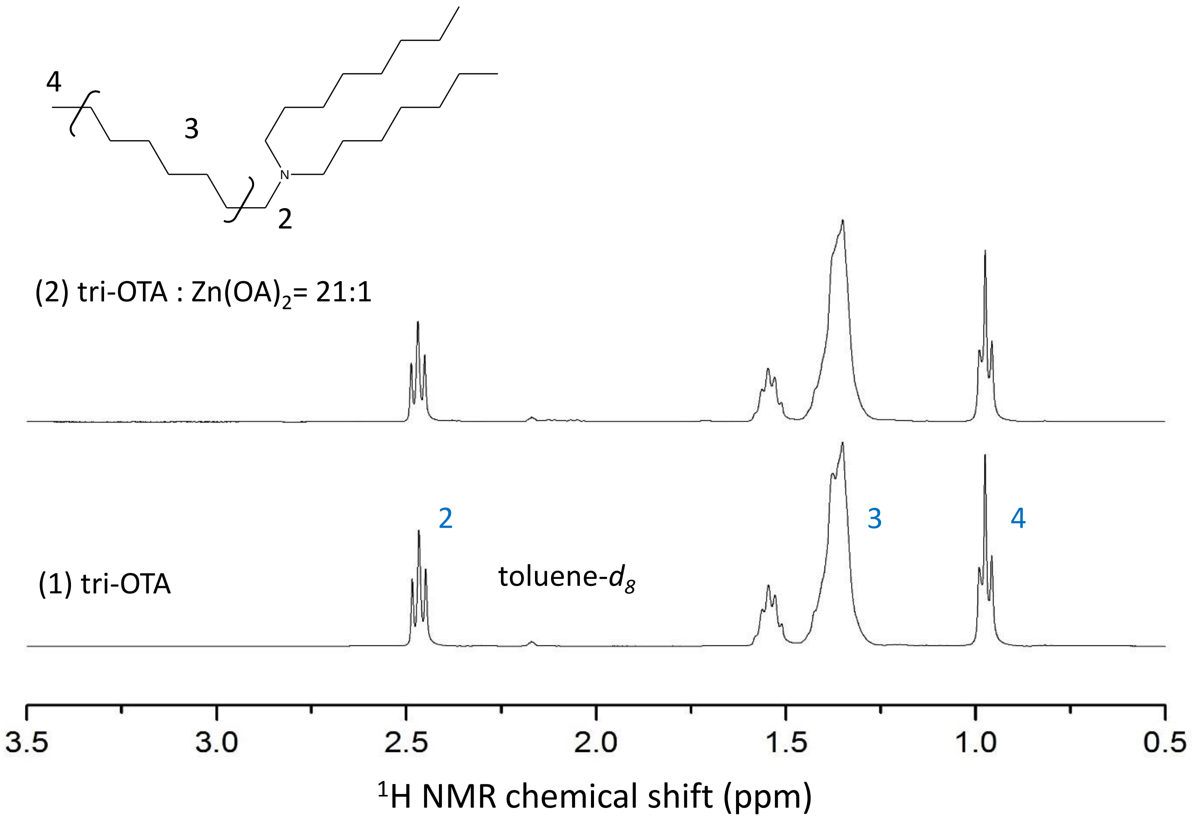

Figure S6-4b. Preliminary ^{1}H NMR spectra collected to help us understand the role of a primary amine added in the structural transformation from IP-299 to MSC-299, with top and bottom panels for di-OTA (98%, Aldrich) and tri-OTA (98%, Aldrich), respectively.

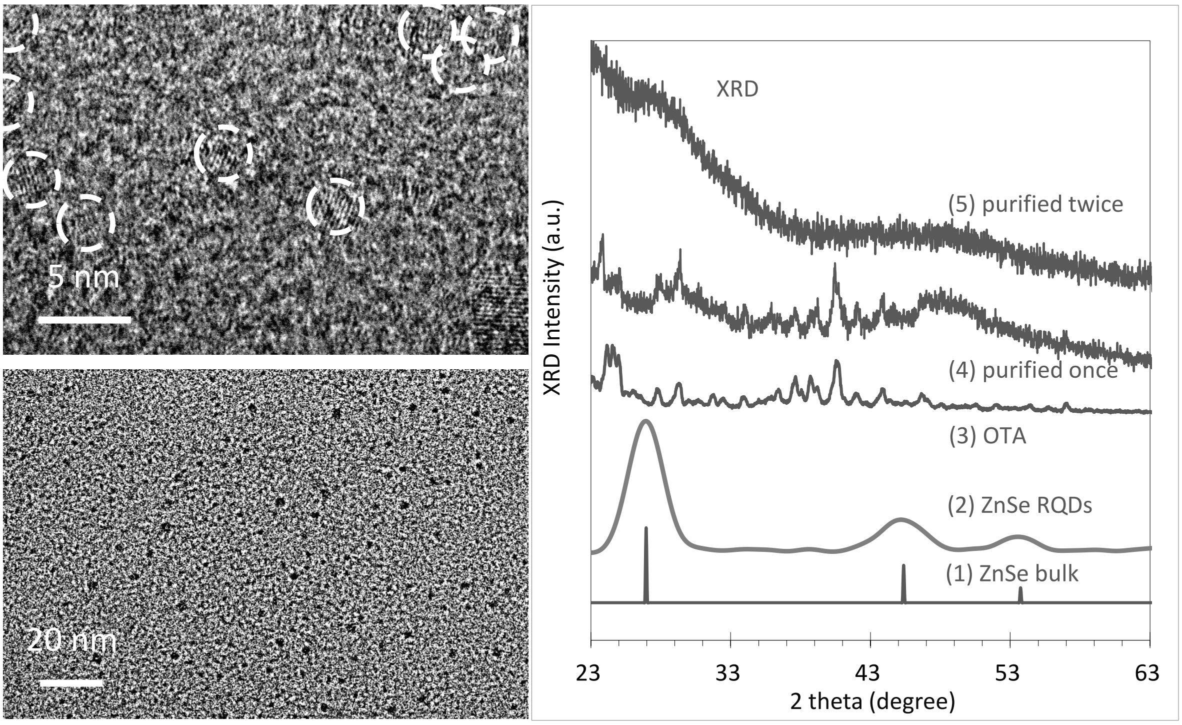

Figure S6-5a. TEM images (left) of the MSC-299 (purified once) and X-ray powder diffraction (XRD) patterns (right) of (1) the zinc-blende ZnSe bulk,[1] (2) ZnSe RQDs,[1] (3) OTA powder, (4) ZnSe MSC-299 purified once, and (5) ZnSe MSC-299 purified twice. The stability of MSC-299 seemed to be challenged with twice purification. The TEM images were taken using JEM-2100F operating at 200 kV. The XRD patterns were collected using a Shimadzu X-ray diffractometer 6100 (with Cu Kα (λ = 1.5418 Å)) at room temperature between 10 and 70° (for ZnSe MSC-299 samples) and between 15 and 80° (for ZnSe RQDs and bulk) in the 2ϑ mode at a rate of 1° per minute; here, we only show the 2ϑ range from 23 to 63°. For the sample purification, one as-synthesized mixture (200 μL) was dispersed in a mixture of 1.0 mL of OTA and 0.8 mL of acetonitrile (CH_3CN, 99.8%, Aldrich). Centrifugation was then carried out (8000 rpm/min, 3 min on Shuke Centrifuge TG-16S). For the second purification, the precipitate from first purification was dissolved in a mixture of 0.5 mL of Tol and 0.8 mL of acetonitrile followed by the centrifugation (8000 r/min, 3 min). It is noteworthy that the conventional characterization tools (including TEM[2,3] and XRD here) are not able to provide the accurate size and structure for the ZnSe MSCs studied. Although the formula and stoichiometry is an important piece of information, cluster stoichiometry seems to be related to the structure of a cluster. A stoichiometric composition has been proposed for a

core-cage structure[4] and a non-stoichiometric composition for a tetrahedral structure.[5] Meanwhile, the cluster mass measurement is still full of uncertainty, such as the amount of surface ligands before and after the measurement (LDI and MALDI, for example).

[1] K. Yu, A. Hrdina, X. Zhang, J. Ouyang, D. M. Leek, X. Wu, M. Gong, D. Wilkinson, C. Li, *Chem. Commun.* **2011**, *47*, 8811.

[2] M. Zanella, A. Z. Abbasi, A. K. Schaper, W. J. Parak, *J. Phys. Chem. C* **2010**, *114*, 6205.

[3] L. Xie, Y. Shen, D. Franke, V. Sebastian, M. G. Bawendi, K. F. Jensen, *J. Am. Chem. Soc.* **2016**, *138*, 13469.

[4] A. Kasuya, R. Sivamohan, Y. A. Barnakov, I. M. Dmitruk, T. Nirasawa, V. R. Romanyuk, V. Kumar, S. V. Mamykin, K. Tohji, B. Jeyadevan, K. Shinoda, T. Kudo, O. Terasaki, Z. Liu, R. V. Belosludov, V. Sundararajan, Y. Kawazoe, *Nat. Mater.* **2004**, *3*,99.

[5] A. N. Beecher, X. Yang, J. H. Palmer, A. L. LaGrassa, P. Juhas, S. J. L. Billinge, J. S. Owen, *J. Am. Chem. Soc.* **2014**, *136*, 10645.

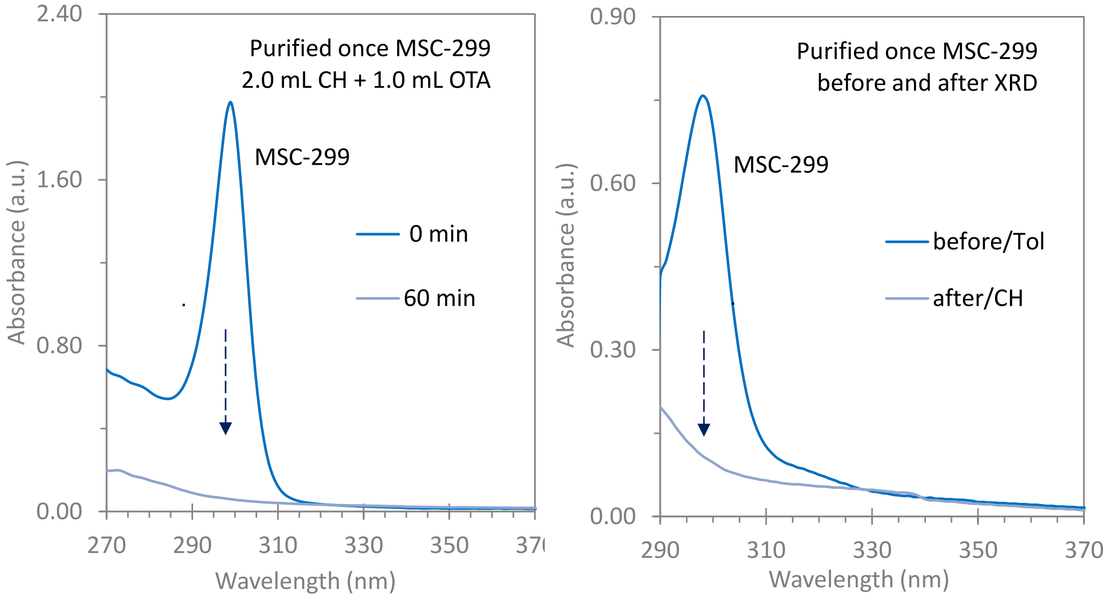

Figure S6-5b. Absorption spectra of the ZnSe MSC-299 with once purification (a) before (blue trace, in Tol) and after (light blue trace, in CH) the XRD data collection shown in Figure S6-5a (right); (b) dispersed in 2.0 mL CH - 1.0 mL OTA at 0 min (blue trace) and 60 min (light blue trace). The ZnSe MSC-299 seemed to have limited stability and changed during XRD measurments.

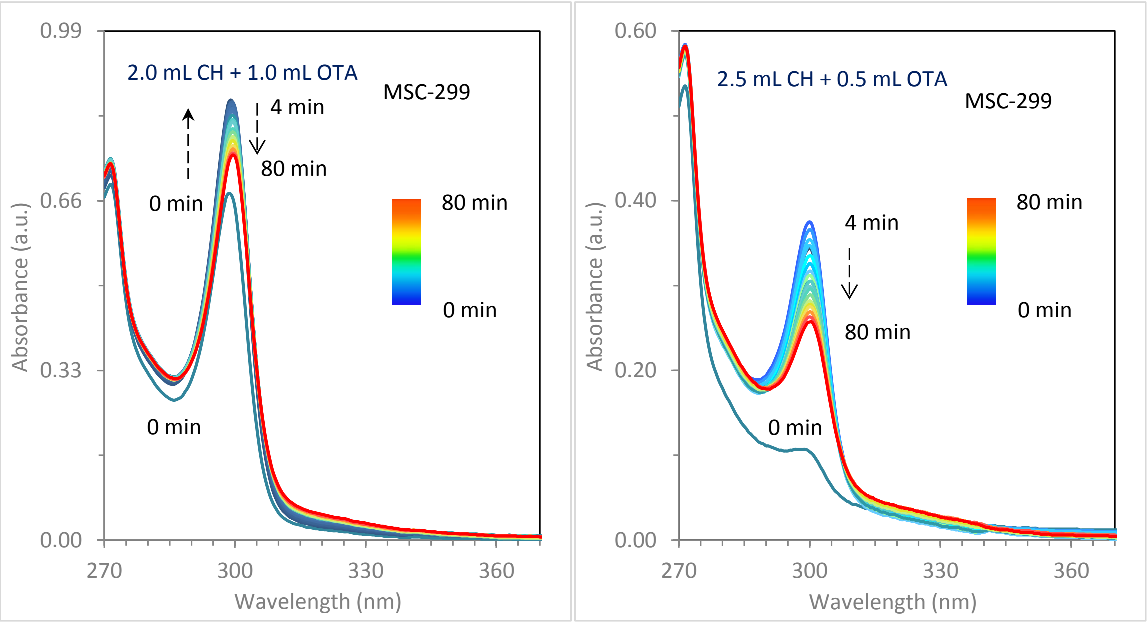

Figure S6-6. The optical study of the 160 °C/30 min sample (30 μL), which was extracted from the 4Zn(OA)$_2$ + 1SeTOP + 4HPPh$_2$ reaction and then was dispersed in the 3.0 mL mixtures containing 1.0 mL (left) and 0.5 mL (right) of OTA (as indicated). The spectra were collected every 4 minutes up to 80 minutes. In the first 4 minutes, there was an increase of the ZnSe MSC-299 population detected. Afterwards, the MSC-299 population decreased. The stability of MSC-299 seems to be affected by the nature and amount of the amine used in the dispersion. Also, the stability of purified MSC-299 decreased, which can be attributed to the lose of surface ligands during purification processes (Figure S6-5b).

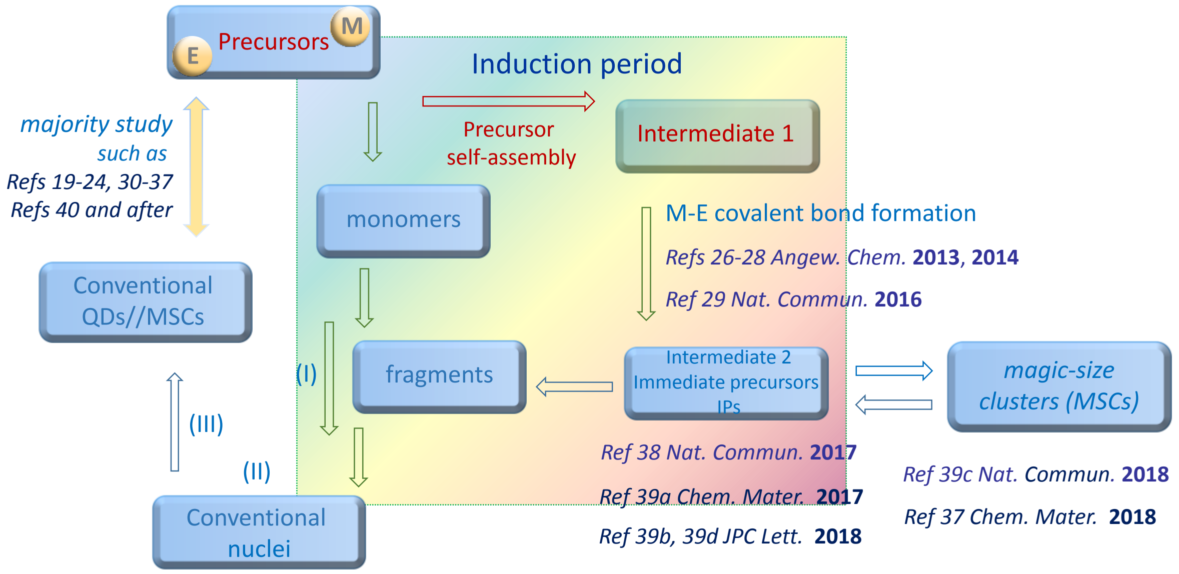

Scheme S1. Schematic drawing showing how the present study conceptualizes the induction period, which has been acknowledged to be rather a black box by the community, such as in a private communication by Dr. J. S. Steckel, the first author of Ref 24 when he was Head of Chemistry Division, QD Vision. Via a two-step approach, the immediate precursor (IP) of MSCs can be synthesized in the first step before the conventional QDs can form, due to precursor self-assembly that results in a dense-phase reaction. By a side note, Symbols (I) to (III) represent the three stages of Classical Nucleation Theory (CNT) illustrated by the LaMer model (V. K. Lamer, R. H. Dinegar, *J. Am. Chem. Soc*. **1950**, *72*, 4847). When the monomer amount increases (I) to a certain degree (II, above saturation), burst of nucleation takes place resulting the decrease of the monomer amount and the growth of particles (III). The present study (as highlighted in red), together with Refs 38 and 39, enriches our understanding of Stage (I), actually. Furthermore, Ref 37 and Ref 39c study the self-assembly and isomerization of MSCs, respectively. Refs 26 to 29 examine the general foramtion pathway of M-E colvanet bonds from the starting M and E precursors; the bond formation chemistry also results in monomers; the foramtion process of RQDs and MSCs is similar to polymerization. Ref 38 explores the existence of the immediate precursor (IP) of MSCs, while Ref 39a demonstrates that the isomerization from IPs to MSCs follows a first-order reaction kinetics behavior. Ref 39b addresses the existence of the MSC to IP to fragment to RQD pathway, while Ref 39d discusses about slowing down the IP to MSC transformation.

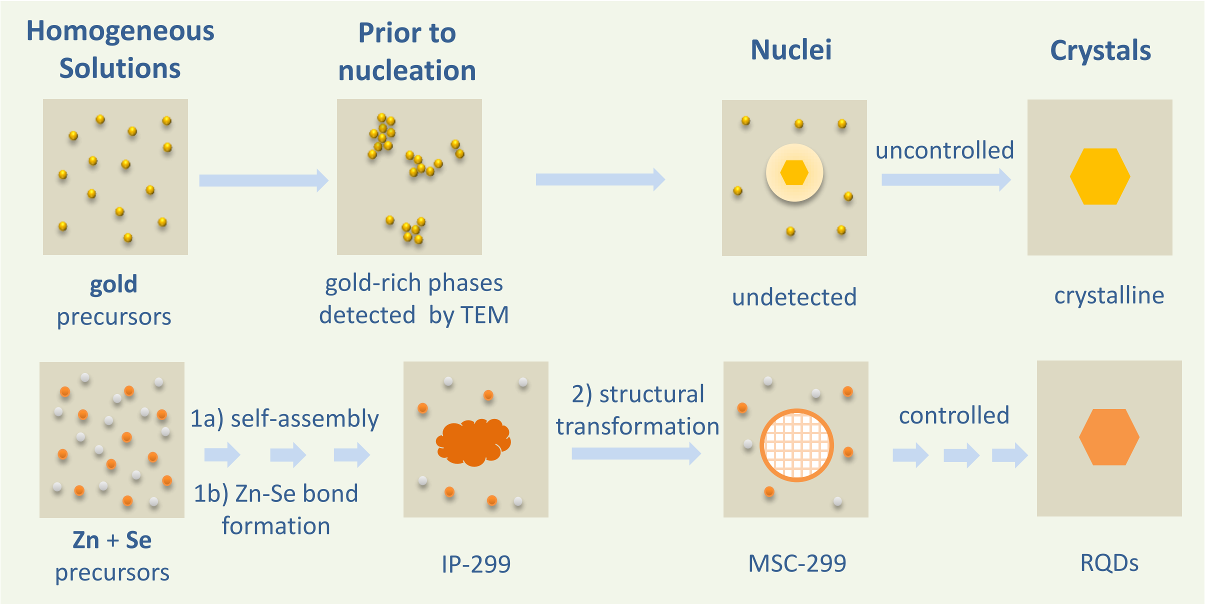

Scheme S2. Schematic drawing for the comprehension of the two-step nucleation based on a TEM study (top panel)[1] and on optical measurements of the present system (bottom panel). It is of help to point out that, in addition to noble metal,[1] the two-step nucleation model has been explored for calcium phosphate and carbonate.[2-8] On the way to the formation of nuclei from a homogenous solution, the non-classical nucleation theory, namely the two-step nucleation theory suggests that density and structure is decoupled, with an increase in density followed by the development of crystallinity.[1-8] However, these systems did not provide experimental evidence on the second step for the formation of nuclei. The present work on the structural transformation of IP-299 to MSC-299 does provide the very experimental evidence for the second step. MSC-299 is a nucleus without further growth. And the insight gained on the self-assembly process leading to IP-299 embraces the advance of MNT for the first step.

[1] N. D. Loh, S. Sen, M. Bosman, S. F. Tan, J. Zhong, C. A. Nijhuis, P. Král, P. Matsudaira, U. Mirsaidov, *Nat. Chem.* **2017**, *9*, 77.
[2] P. G. Vekilov, *Cryst. Growth Des. 2010*, *10*, 5007.
[3] A. Dey, P. H. H. Bomans, F. A. Müller, J. Will, P. M. Frederik, W. Gijsbertus, N. A. J. M. Sommerdijk, *Nat. Mater.* **2010**, *9*, 1010.
[4] W. J. E. M. Habraken, J. Tao, L. J. Brylka, H. Friedrich, L. Bertinetti, A. S. Schenk, A. Verch, V. Dmitrovic, P. H. H. Bomans, P. M. Frederik, J. Laven, P. Schoot, B. Aichmayer, G. With, J. J. DeYoreo, N. A. J. M. Sommerdijk, *Nat. Commun.* **2013**, *4*, 1507.
[5] D. Gebauer, A. Völkel, H. Cölfen, *Science* **2008**, *322*, 1819.
[6] R. Demichelis, P. Raiteri, J. D. Gale, D. Quigley, D. Gebauer, *Nat. Commun.* **2011**, *2*, 590.
[7] M. H. Nielsen, S. Aloni, J. J. D. Yoreo, *Science* **2014**, *345*, 1158.

[8] P. J. M. Smeets, K. R. Cho, R. G. E. Kempen, N. A. J. M. Sommerdijk, J. J. D. Yoreo, *Nat. Mater.* **2015**, *14*, 394.

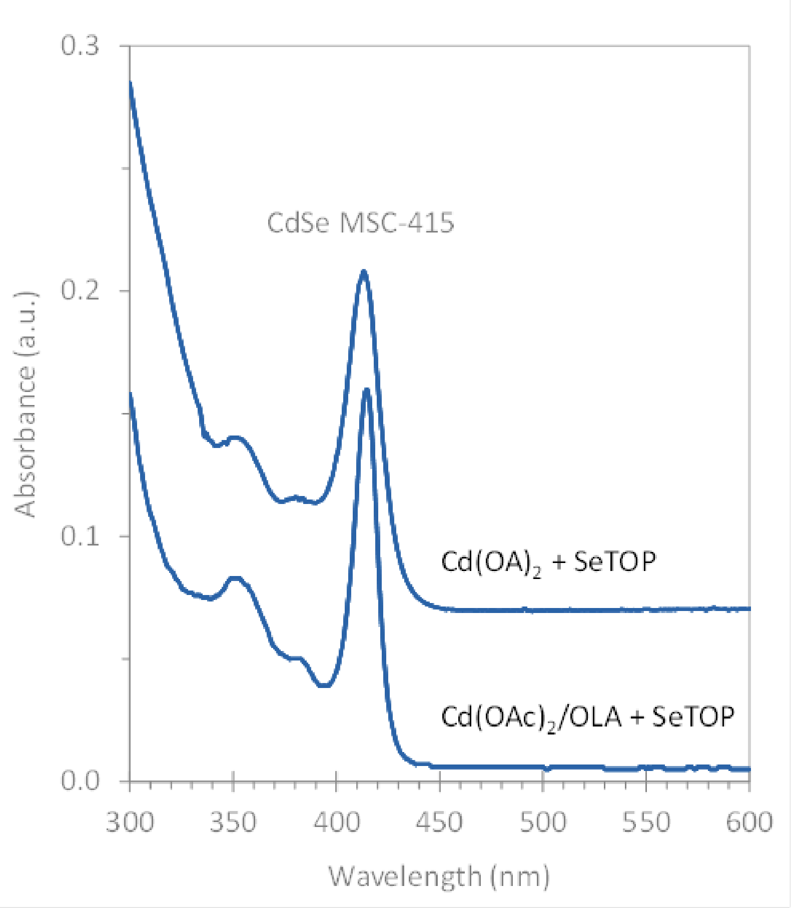

Figure S7. The self-assembly process should be general for the formation colloidal semiconductor MSCs, being independent of the nature of the metal and chalcogenide precursors. Another example is the detection of CdSe MSC-415 from the two reactions of the two different Cd precursors as indicated.

Note S1. On the second step and conventional characterization tools. A couple of outstanding points will be addressed. The role of the primary amine in the IP-299 to MSC-299 transformation is not clearly understood on the molecular level, despite the progress made on the synthesis of the ZnSe MSC-299. Another problem lies in the structural characterization of MSC-299. Accumulated experimental evidence has suggested that the formation of MSCs is surface-related;[1-5] for this reason, we tested a short-chain primary amine, butyl amine (BTA), to replace OTA. Figure S6-3 shows the presence of MSC-299 from the mixture of CH and BTA and not from the mixture of CH and di-BTA. The proton bonded to the N atom in the primary amine is believed to play a critical role in the transformation of IP-299 $\Rightarrow$ MSC-299 (see our ^{1}H NMR study by Figure S6-4), similar to that for CdTe MSC-371.[1] However, we have no full understanding of the role of a primary amine at the molecular level, and the precise mechanism remains an open question.

At the same time, the structural characterization of MSCs has not been unambiguous. For example, in a similar system CdSe MSCs exhibiting absorption peaks at 415 nm[6] or 408 nm,[7] there is no consensus in the literature regarding the structure and stoichiometry composition; two different structures were reported, a core cage structure with 1Cd-to-1Se stoichiometry[6] and a tetrahedral structure with off 1Cd-to-1Se stoichiometry.[7] Transmission electron microscopy (TEM) does not provide an accurate measurement of the diameters of small-size NCs,[8,9] including MSCs; the underlying reasons may be related to changes occurring in the clusters, such as during TEM sample preparation.[10] Figure S6-5a shows the TEM images and X-ray diffraction (XRD) of MSC-299. Structural characterization is further inhibited by the limited stability of MSCs, which is affected by several factors (including the nature and amount of primary amines used) and the interplay between them (Figures S6-5b and S6-6). Thus, no reliable structural information can be obtained using XRD. Even if our samples had been stable (under the experimental condition for XRD), the use of "phase" to describe MSC structures is not entirely appropriate, due to the very small size of the clusters.[2,12] MS characterizations also provide limited information for MSCs (regarding their surface ligands and core compositions).[1,2,6,9,13,14] Given the unsuitability of MSCs for such characterization tools,[1,2,6,8,9,13,14] an uncertainty remains regarding the structure and composition of MSCs. Hence, we see there is a need to revisit relevant characterizations and to develop suitable characterization tools as well as sophisticated theoretical models for the

structure and property of MSCs.[15]

[1] M. Liu, K. Wang, L. Wang, S. Han, H. Fan, N. Rowell, J. A. Ripmeester, R. Renoud, F. Bian, J. Zeng, K. Yu, *Nat. Commun.* **2017**, *8*, 15467.

[2] a) T. Zhu, B. Zhang, J. Zhang, J. Lu, H. Fan, N. Rowell, J. A. Ripmeester, S. Han, K. Yu, *Chem. Mater.* **2017**, *29*, 5727; b) J. Zhang, X. Hao, N. Rowell, T. Kreouzis, S. Han, H. Fan, C. Zhang, C. Hu, M. Zhang, K. Yu, *J. Phy. Lett*. **2018**, *9*, 3660; c) B. Zhang, T. Zhu, M. Ou, N. Rowell, H. Fan, J. Han, L. Tan, M. T. Dove, Y. Ren, X. Zuo, S. Han, J. Zeng, K. Yu, *Nat. Commun*. **2018**, *9*, 2499; d) D. Zhu, J. Hui, N. Rowell, Y. Liu, Q. Chen, T. Steegemans, H. Fan, M. Zhang, K. Yu, *J. Phy. Chem. lett*. **2018**, *9*, 2818.

[3] K. Yu, J. Ouyang, D. M. Leek, *Small* **2011**, *7*, 2250.

[4] C. Landes, M. Braun, C. Burda, M. A. El-Sayed, *Nano Lett*. **2001**, *1*, 667.

[5] D. C. Gary, M. W. Terban, S. J. L. Billinge, B. M. Cossairt, *Chem. Mater*. **2015**, *27*, 1432.

[6] A. Kasuya, R. Sivamohan, Y. A. Barnakov, I. M. Dmitruk, T. Nirasawa, V. R. Romanyuk, V. Kumar, S. V. Mamykin, K. Tohji, B. Jeyadevan, K. Shinoda, T. Kudo, O. Terasaki, Z. Liu, R. V. Belosludov, V. Sundararajan, Y. Kawazoe, *Nat. Mater.* **2004**, *3*, 99.

[7] A. N. Beecher, X. Yang, J. H. Palmer, A. L. LaGrassa, P. Juhas, S. J. L. Billinge, J. S. Owen, *J. Am. Chem. Soc*. **2014**, *136*, 10645.

[8] M. Zanella, A. Z. Abbasi, A. K. Schaper, W. J. Parak, *J. Phys. Chem. C* **2010**, *114*, 6205.

[9] L. Xie, Y. Shen, D. Franke, V. Sebastian, M. G. Bawendi, K. F. Jensen*, J. Am. Chem. Soc*. **2016**, *138*, 13469.

[10] Y. Liu, B. Zhang, F. Fan, N. Rowell, M. Willis, X. Zheng, R. Che, S. Han, K. Yu, *Chem. Mater.* **2018**, *30*, 1575.

[12] A. Proykova, R. Berry, *J. Phys. B: At. Mol. Opt. Phys*. **2006**, *39*, R167.

[13] Y. Wang, Y. H. Liu, Y. Zhang, F. Wang, P. J. Kowalski, H. W. Rohrs, R. A. Loomis, M. L. Gross, W. E. Buhro, *Angew. Chem. Int. Ed. Engl.* **2012**, *51*, 6154.

[14] F. Muckel, J. Yang, S. Lorenz, W. Baek, H. Chang, T. Hyeon, G. Bacher, R. Fainblat, *ACS Nano* **2016**, *10*, 7135.

[15] J. Lee, J. Yang, S. G. Kwon, T. Hyeon, *Nat. Rev. Mater*. **2016**, *1*, 1.
